# Application of Pauson–Khand reaction in the total synthesis of terpenes

**DOI:** 10.1039/d1ra05673e

**Published:** 2021-11-29

**Authors:** Majid M. Heravi, Leila Mohammadi

**Affiliations:** Department of Chemistry, School of Physics and Chemistry, Alzahra University Vanak Tehran Iran mmh1331@yahoo.com mmheravi@alzahra.ac.ir +98 2188041344 +98 9121329147

## Abstract

The Pauson–Khand reaction (PKR) is a formal [2 + 2 + 1] cycloaddition involving an alkyne, an alkene and carbon monoxide mediated by a hexacarbonyldicobaltalkyne complex to yield cyclopentenones in a single step. This versatile reaction has become a method of choice for the synthesis of cyclopentenone and its derivatives since its discovery in the early seventies. The aim of this review is to point out the applications of PKR in the total synthesis of terpenes.

## Abstract 2

The Pauson–Khand reaction (PKR) is a formal [2 + 2 + 1] cycloaddition involving an alkyne, an alkene and carbon monoxide mediated by a hexacarbonyldicobaltalkyne complex to yield cyclopentenones in a single step.

## Graphical abstract

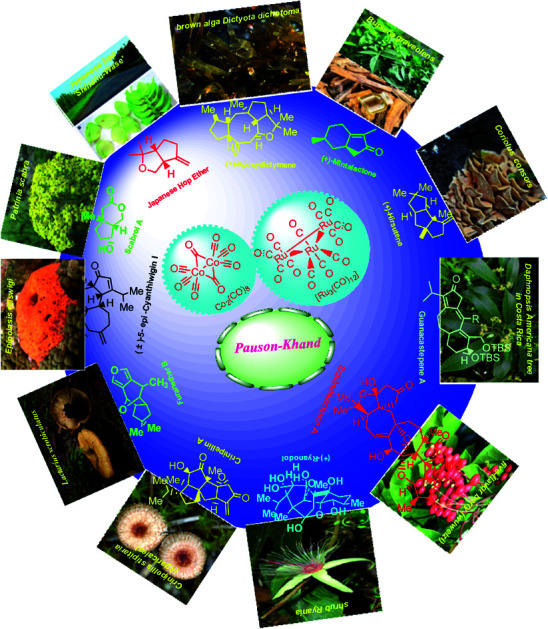

## 1. Introduction

**Scheme 1:**
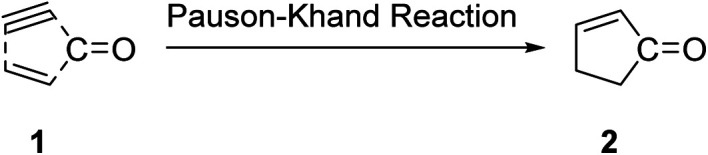
Formation of three new bonds in PKR resulting in the formation of cyclopentanones.

The Pauson–Khand reaction (PKR or PK-type reaction) is formally a chemical reaction in which a triple bond, a double bond and carbon monoxide are subjected to a [2 + 2 + 1] cycloaddition reaction to form an α,β-cyclopentenone.^1^ This entails the formation of three new bonds and one or two rings in the intermolecular or intramolecular fashion, respectively (Scheme 1).^1^

**Scheme 2:**
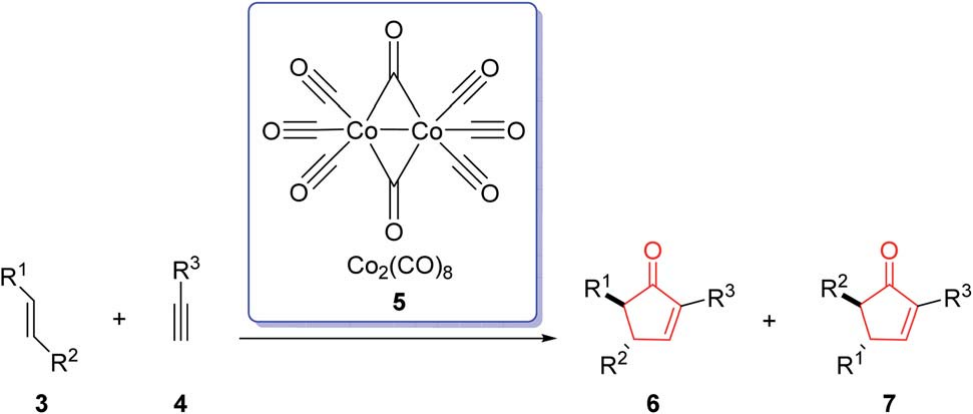
The first example of Pauson–Khand reaction without regioselectivity.

The first example of this reaction was reported in 1973.^2^ In this reaction, norbornene reacted with the phenyl acetylene–hexacarbonyldicobalt complex to afford the corresponding cyclopentenone in 45% yield by using a stoichiometric amount of dicobalt octacarbonyl [Co_2_(CO)_8_] (Scheme 2).^3^ This reaction was discovered by Ihsan Khand (1935–1980), who was working as a postdoctoral fellow with Peter Pauson (1925–2013),^4^ at the University of Strathclyde in Glasgow.

**Scheme 3:**
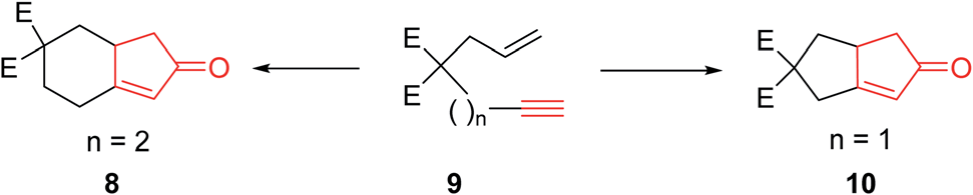
Regioselective intramolecular PKR.

The original PKR had several drawbacks such as only being proceeded in the presence of stoichiometric amount of dicobalt octacarbonyl (Co_2_(CO)_8_) as the only cluster, performed thermally under relatively harsh reaction conditions which resulted in desired transformations but with low efficiency. It also showed limited substrate scope covering narrow range of substrates. The use of strained olefins was necessary to obtain acceptable yields. In addition, the reactions typically afforded a mixture of regioisomers if unsymmetrical alkynes and alkenes were used. In several cases, the PKRs showed poor conversions and especially selectivities (chemo-, regio- and stereoselectivities). Therefore, an important breakthrough was required which was obtained by Schore and co-workers,^5,6^ who reported that carbon-tethered enyne precursors can be subjected to an intramolecular Pauson–Khand reaction (IPKR) in good yields with high regioselectivity. In other words, it was not necessary to use strained olefins as starting materials. In 1983, Schore and coworkers achieved and demonstrated the intramolecular PKR (IPKR).^6^ A series of 5,5-disubstituted enynes 9 were subjected to PKR conditions to obtain 5,6-used bicycles 8 or 10 under PKR (thus, IPKR) conditions its good efficiency and conversions^7^ (Scheme 3).^5^

The PKR has received much attention of chemical community, especially synthetic organic chemists, as a method of choice for scientific studies because of the increase in diversity of the available starting materials. Therefore, it has rapidly improved and developed during these since its discovery.^8–15^

As an example an essential contribution to improvement of the promotion of PKR Smit and Caple in 1986.^16^ They immobilized the reagents onto various solid supports. In these cases, PKRs were performed at lower temperatures and completed in shorter reaction times^16^ [although other metals were found to catalyze the PKR, use of Co_2_(CO)_8_ in stoichiometric amount showed several merits. The Co_2_(CO)_8_ complex is inexpensive and commercially available, it tolerates a wide spectrum of functional groups, exhibits activity toward both terminal and internal alkynes].

**Scheme 4:**
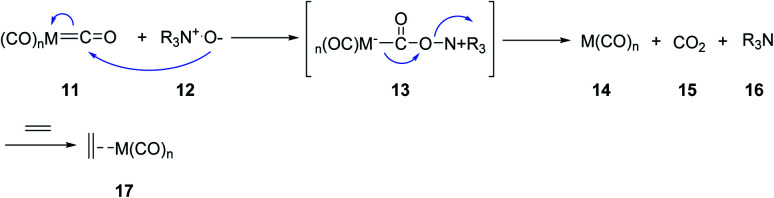
Formation of a vacant site in the cobalt cluster.

Jeong *et al.*^17^ Schreiber and coworkers independently circumvented other problems such as requirement of high temperatures and CO pressures, as well as long reaction times.^18^ They found that trimethylamine *N*-oxide and *N*-methylmorpholine *N*-oxide can accelerate the PKR dramatically. A wide range of cyclopentenones were obtained in good yields even at room temperature in the presence of *N*-oxides. It is presumed that *N*-oxides affect the reaction *via* oxidative liberation of the CO ligands of the metal complex facing up a coordination site. Thus, the following oxidative alkene addition becomes, the rate-determining step, accelerating the PKRs (Scheme 4). Other reagents were also found to assist PKRs to proceed smoothly and rapidly; there include silica gel,^13,16^ molecular sieves,^19,20^ alkyl methyl sulfides,^21^ and primary amines.^22^

**Scheme 5:**
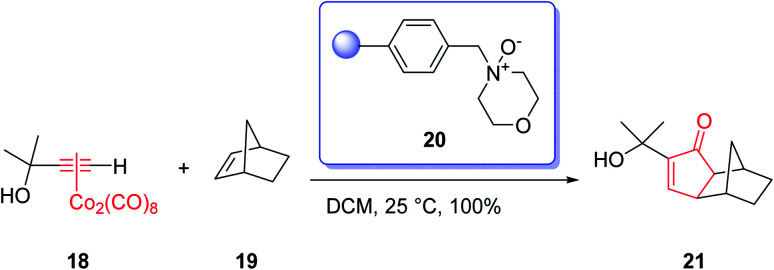
A PKR performed on solid state.

The most commonly used amine *N*-oxides are trimethylamine *N*-oxide (TMANO) and *N*-methylmorpholine *N*-oxide (NMO). The latter has recently been immobilized onto a solid support which facilitates the work-up of the reaction and results in excellent yields of the products of PKRs (Scheme 5).^23^

**Scheme 6:**
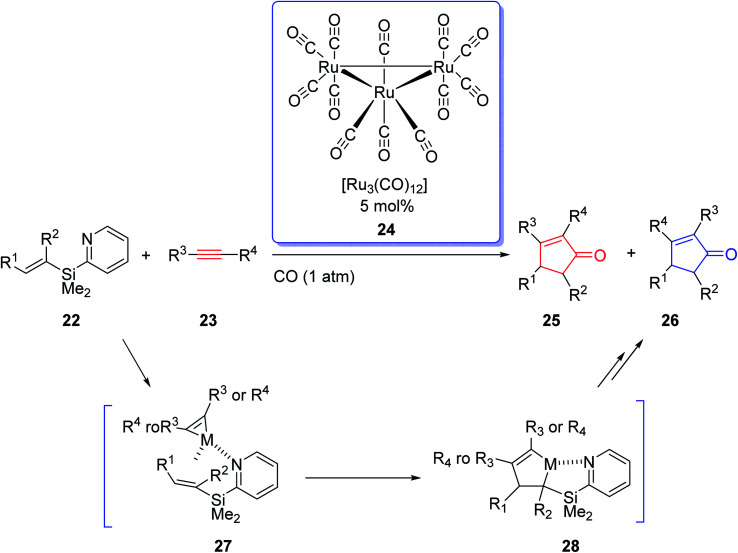
The catalytic intermolecular Pauson–Khand reaction directed by a pyridylsilyl group.

Due to the innovation of IPKR, bicyclic frameworks which are prevalent in naturally occurring compounds can be constructed in one step *via* IPKR. Therefore, IPKR has found several applications in the total synthesis of natural products frequently used as the key step.^6,24–26^ Synthetic organic chemistry has developed momentously owing to the exponential growth in transition-metal-catalyzed reactions. The effect of transition-metal-catalyzed reactions on organic synthesis could be evaluated by the wide range of molecules that have been developed from simple compounds to complex natural products. In 2002, J. Yoshida and coworkers intelligently expanded the metal-catalyzed organic transformations to PKR.^27^ They used the Ru carbonyl complex [Ru_3_(CO)_12_] 24 in catalytic amount (0.5 mol%) in the reaction of olefins containing an easily removable pyridisilyl group, readily obtained from alkynes under one atmosphere of CO. This metal catalyzed-PKR promotes the reaction efficiently since the pyridyl group directs it to proceed *via* the coordination of the nitrogen with the metal that provided complete regioselectivity with unsymmetrical olefins (Scheme 6).^27^

These achievements encouraged several research groups to investigate the scope and limitations of using other metals as catalyst in PKR. In this regard, various alkenes, alkynes, and carbon monoxide in the presence of different transition metals under PKR conditions were converted into the corresponding cyclopentenone derivatives.^28,29^ For this purpose, various carbonyl complexes of iron (iron pentacarbonyl),^29–32^ tungsten (tungsten pentacarbonyl),^32^ chromium, molybdenum (molybdenum hexacarbonyl),^33^ heterobimetallic cobalt/tungsten complexes,^34^ and cobalt complex created *in situ* from alkyne cobalt complex and triethylsilane,^35^ titanocene complexes,^34,36–38^ Co_2_(CO)_8_ with high-intensity visible light system,^39^ highly purified Co_2_(CO)_8_,^40^ and other ruthenium complexes^35,38,41,42^ were successfully used under PKR conditions.^43–46^ Noticeably, the above-mentioned metal-catalyzed IPKR reactions had to be performed under medium or high pressure of carbon monoxide. In addition to ruthenium, rhodium complexes efficiently catalyze PKR which has attracted considerable attention. In 2002, Jeong and co-workers,^47^ successfully used several rhodium complexes in various reactions under PKR conditions. Noticeably, some need activation with AgOTf prior to be used. After the appearance of the first IPKR in 1984,^16,48^ a plethora of papers were published regarding the successful catalytic IPKR.^2,49–51^

In the nineties, several other developments in the PKR were achieved, including the introduction of an asymmetric variant (APKR). In general, bicyclic cyclopentenones as PKR products are valuable synthetic targets. The PKR is a powerful tool for the construction of such structural units. On the other hand, bicyclic cyclopentenones are prevalent scaffolds in natural products. Therefore, the asymmetric variant of PKR (APKR) is a unique and useful technique for the stereoselective construction of bicyclic cyclopentenones as a key step in the multistep total synthesis of natural products. Shibata and coworkers, reported the first example of a catalytic APKR in 2000.^29^

Their strategy was based on a chiral *ansa*-metallocene complex of titanium. Subsequently, other catalytic APKRs were supplemented by a plethora of promising reports, comprising chiral catalysts derived from binaphthyl phosphines and iridium, cobalt^33,52^ or rhodium precatalysts.^32^

The efficiency of enantioselectivity of APKR mainly depends on the selection of correct substrates to induce asymmetry in the PKR. For this purpose, four approaches are available (a) using chiral substrate; (b) employing appropriate chiral auxiliary (c) using chiral metal complex and (d) the chiral promoter. The best results have been obtained using chiral substrates or chiral auxiliaries. Although, the use of chiral metal complex to give the best results, but it is still in the beginning stages and will be developed in a way to give excellent results expectedly in the future.

During investigation on the Rh-catalyzed PKR, two chiral catalysts were reported for APKR under an atmospheric pressure of CO. The chiral catalysts were (*S*)-BINAP/Co_2_(CO)_8_,^33,52^ (*S*)-tolBINAP/[Ir(cod)Cl]_2_ (ref. 29) and (*S*)-BINAP/[RhCl(CO)_2_].^2,31,32^

### 1.1. Mechanism of Pauson–Khand reaction

**Scheme 7:**
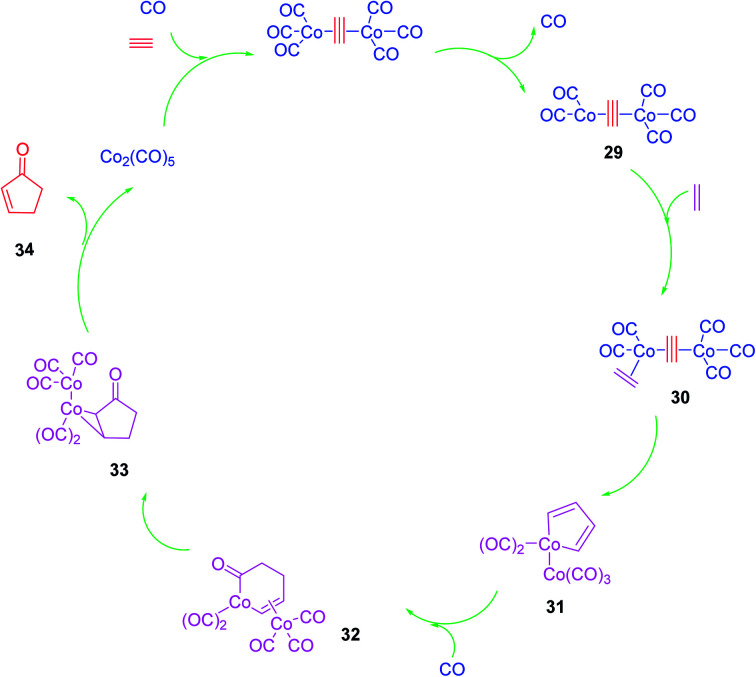
A plausible mechanistic proposal for the Pauson–Khand reaction.

Although the Pauson–Khand reaction was discovered in 1973, a plausible mechanism for it was proposed by Magnus *et al.* in 1985 as illustrated in (Scheme 7).^53^ Nowadays, this mechanism is believed to be undisputable since it has recently been confirmed by detailed negative ion electrospray collision testing.^54^ Accordingly, the PKR is believed to commence with the generation of the alkyne-Co_2_(CO)_8_ complex 29, lans of a carbonyl ligand to vacate a coordination site, olefin coordination 30, followed by insertion, occurring at the end of the alkyne, which is less hindered to produce *in situ* the metallacycle 31. The latter reacts promptly with inserted CO ligand to produce complex 32 followed by reductive elimination of 33 proceeds to furnish the desired target, cyclopentenone 34. It is noteworthy that all the bond-forming steps took place on just one cobalt atom. The other cobalt atom which is present in the complex is supposed to function as an anchor which has extra electronic effects on the bond-forming metal atom *via* the present metal–metal bond^55^ (Scheme 7).^53^

Although the prominence and importance of all kinds of PKR have been extensively covered by the previously published reviews,^56,57^ its applications in total synthesis of natural products have largely been overlooked and limited to a subsection that mentions some total syntheses using PKR. These natural products are vincristine (Oncovin), Navelbine (vinorelbine), etoposide (VP-16), teniposide (VM-26), Taxol (paclitaxel) and most recently in 1996, Taxotere (docetaxel), topotecan (Hycamtin), sexestobergsterol, spatane, daphne, iridomirmecin, dendrobin, kalmanol and β-cuparenone were mentioned.^58,59^

## 2. Applications of Pauson–Khand reaction in the total synthesis of terpenes

### 2.1. Monoterpenes

In 1968, Naya and co-workers initially isolated ether 50 which is a monoterpene from the Japanese hop “Shinshu-Wase”.^60^ This naturally occurring compound is also present in Spalter hops.^61^ Fascinatingly, it is thought to contribute to both the taste and aroma in low concentrations in a number of beers.^62–66^ In 2016, Park research group produced Japanese hop extracts employing a standardized method.^67^ This method facilitates the production of improved diagnostic and immunotherapeutic reagents.^67^

**Scheme 8:**
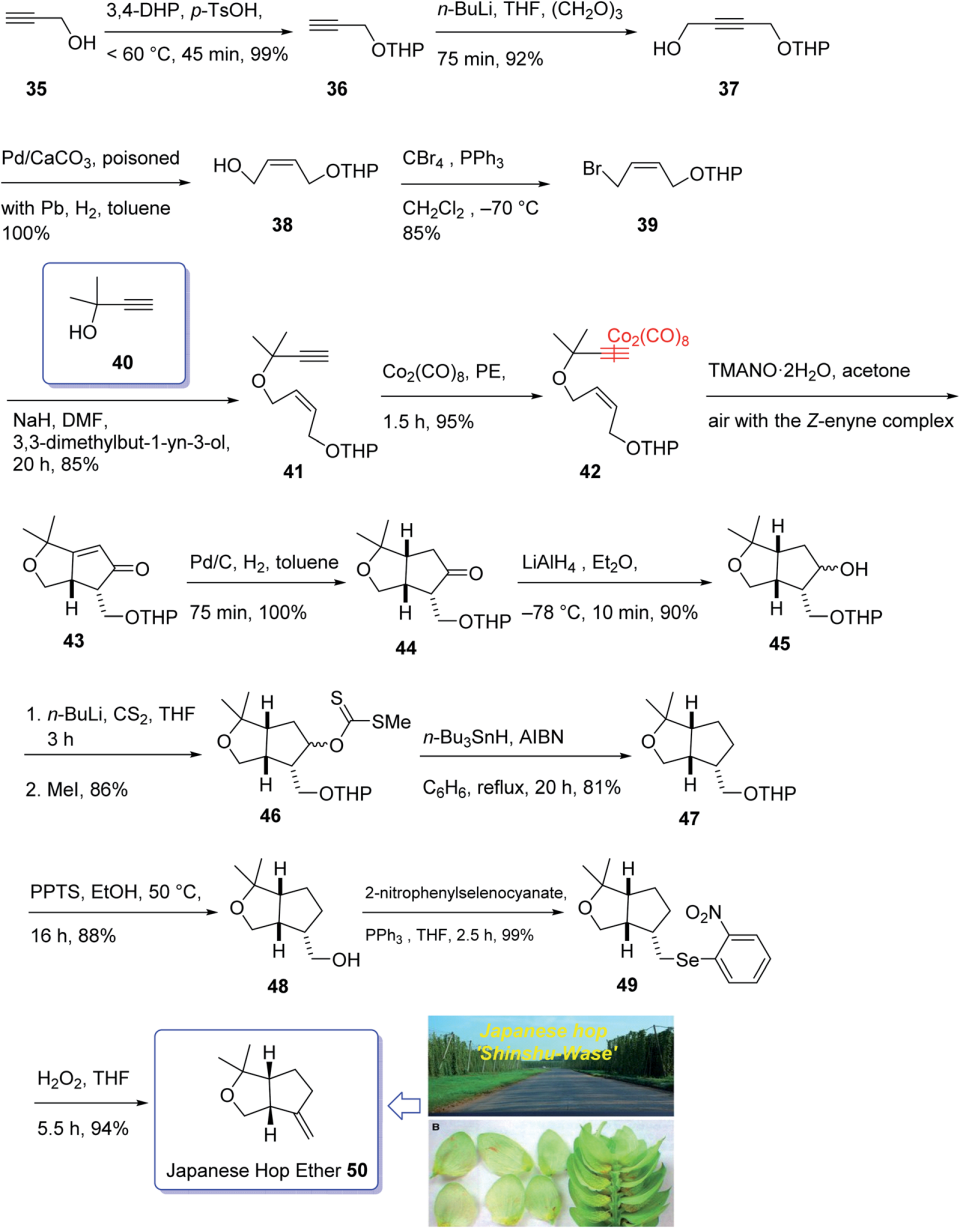
Total synthesis of Japanese hop ether 50.

The first reported total synthesis of Japanese hop ether was reported by Imagawa *et al.* in 1979 which was claimed unreliable^65,68^ and since that time few alternative pathways have also been claimed.^69^ In 2005, Kerr *et al.* achieved and reported a concise (fourteen steps) total synthesis of monoterpene Japanese hop ether in 29% overall yield. They used an intramolecular Pauson–Khand reaction under mild *N*-oxide promoted conditions and with complete retention of alkene configuration (for both *cis*- and *trans*-alkenes) in the product cyclopentenone, as the crucial key step.^65^ They started with propargyl alcohol 35 and transformed it to the corresponding THP-protected derivative 36 in almost quantitative yield. The latter upon deprotonation, using *n*-BuLi, with subsequent reaction with solid *para*-formaldehyde under ultrasound irradiation furnished the mono-protected ynediol 37 in excellent yield. The *Z*-olefin 38 was obtained in virtually quantitative yield by hydrogenation of ynediol 37 over Lindlar's catalyst and the obtained allylic alcohol was transformed to the bromide 39 in high yield upon being treated with CBr_4_ and PPh_3_. The bromide 39 was then reacted with the alkoxide anion of dimethylpropargyl alcohol 40 to afford allyl propargyl ether in high yield. In a key step (PKR), compound 41 underwent complexation with dicobalt of a carbonyl to afford 42 in excellent yield. Eventually, the *Z*-enyne complex 42 converted into the product 43 in high yield by use of TMANO·2H_2_O in acetone under air. Having compound 43 available in hand, it was deoxygenated affording 48 after several steps. The latter was then directly converted into organoselenium species 49 upon reaction with *o*-nitrophenylselenyl cyanate and tri-*n*-butylphosphine in excellent yield. The latter was simply treated with H_2_O_2_ permitting the *in situ* generation of the selenoxide, which was subjected to elimination to furnish the desired natural product Japanese hop ether 50 in 94% yield (Scheme 8).^65^

**Scheme 9:**
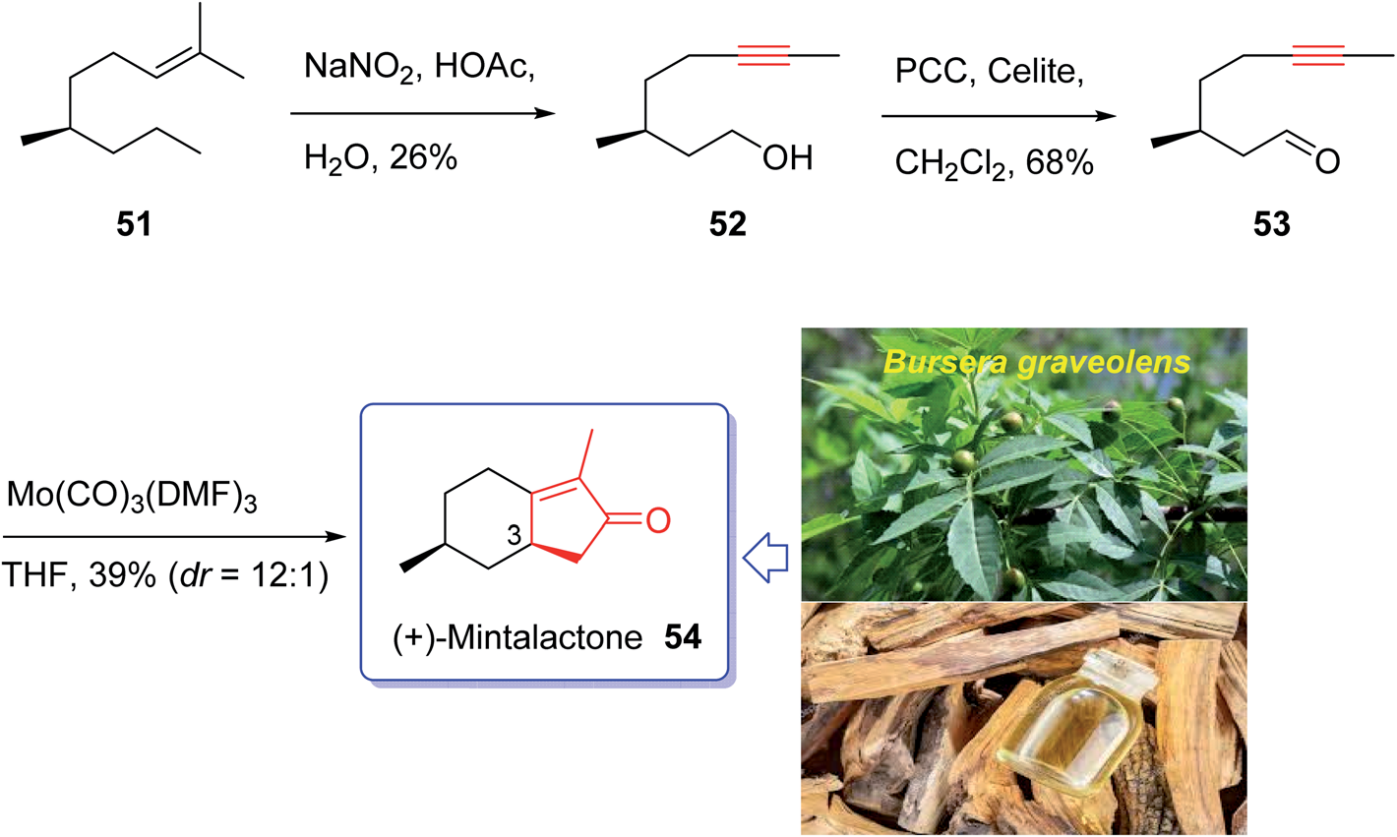
Total synthesis of (+)-mintlactone 54 and (−)-isomintlactone 55.

Iwabuchi and co-workers in 1997,^70^ initially isolated (+)-mintlactone 54 and (−)-isomintlactone 55 as *endo* α,β-unsaturated monoterpene-γ-lactones from the oil of the wood of *Bursera graveolens* (Palo Santo).^70^ In 1968, Muraki and co-workers isolated their enantiomers, *ent*-(−)-54 and *ent*-(+)-55, from *Mentha cardiaca*.^70^ Also, The last two *p*-menthanolides were both isolated from *Mentha arvensis*^71^ and are present as minor constituents of the commercial essential oil.^72^ Interestingly, the total synthesis of the above-mentioned bicyclic monoterpene attracted much attention of synthetic organic chemists.^70,72–78^ Recently, Bates *et al.*^79^ achieved and reported a brief ten-step synthesis of (−)-mintlactone starting from the THP ether of propargyl alcohol *via* a highly asymmetric tin(ii) chloride-catalyzed intramolecular propargylic Barbier reaction with subsequent allenol cyclocarbonylation. Furthermore, Shishido *et al.*^78^ accomplished and reported a concise total synthesis of (−)-mintlactone in ten steps commencing from citronellal. In 2009, Zhai and co-workers^70^ design a pathway for total synthesis of (+)-mintlactone starting from (−)-citronellol 51, using molybdenum-mediated intramolecular hetero-Pauson–Khand reaction as a key step. This pathway started with (−)-citronellol 51 which upon treatment with nitrous acid following Abidi's protocol gave alkynol 52 directly in three steps in 26% overall yield.^80^ The latter was oxidized upon treatment with PCC in CH_2_Cl_2_ at ambient temperature to give ynal 53 as suitable precursor for PKR. The ynal 53 was then treated with freshly prepared organomolybdenum Mo(CO)_3_(DMF)_3_,^81^ as catalyst, in THF at ambient temperature to afford the desirable natural product (+)-mintlactone (54, 39%) along with its inseparable diastereomer, apparently (−)-isomintlactone (55, 3%), in an optimized combined yield of 42%. Interestingly, in this total synthesis, during the PKR, one stereogenic center, two rings, and three covalent bonds (1 C–O and 2 C–C) are generated which proceeded in high diastereoselectivity (C-3, dr = 12 : 1). Due to more stability of a chair conformation (TS-1) in its transition state which theoretically has lower energy than a twist boat one (TS-2), thus (+)-mintlactone 54 must have emerged as a major product. In conclusion, the total synthesis of (+)-mintlactone was accomplished *via* a three-step assemblage which can be exemplified as a new concept in the art of synthetic organic chemistry as “step economy”^82^ and “strategic efficiency”.^83^ Important aspects of this total synthesis involved HNO_2_-induced formal isopropylidene “demethanation” and the Mo(CO)_3_(DMF)_3_-promoted intramolecular PKR (Scheme 9).^70^

The generic name iridoid monoterpenes is derived from the names iridomyrmecin, iridolactone, and iridodial which initially isolated from special species of *Iridomyrmexes*, ants, secreting them as defensive existing either in the glycosidic or in the non-glycosidic form,^84^ these naturally occurring compounds were found the dynamic and active components of folk medicinal plants being used conventionally as medicine for long time as antiviral, antibacterial, and anti-inflammatory.^85,86^ In addition, iridoids are also commercially important since can be used as sex pheromones against some agriculturally important species such as aphids.^87^ Structurally, iridoids contain a confined cyclopenta[*c*]pyran framework as shown for some important members of family. Nevertheless, controlling their stereochemical complexities and the diverse oxygenation patterns, which frequently are confined in *cis*-fused bicycle, are challenging for synthetic organic chemist and render them striking targets for total synthesis. Thus, several fantastic synthetic pathways for their total synthesis have been reported.^88^ Some of them are accomplished and reported by Suh and co-workers^89,90^ and recently one reported by Chakraborty and co-workers.^91^ In spite of the appearance of these reports among the others, a literature survey disclosed few reports on the total synthesis of diverse oxy-functionality pattern observed on the cyclopentane ring of the iridoid framework.^89,92,93^ In 2019, Khan and co-workers reported an efficient and economic strategy to have access to several iridoids (65, 68, 68′, 70, 71, 72, 75, 75′, 79)^94^ using an intramolecular Pauson–Khand reaction (IPKR) as the crucial step to access ten iridoids in a stereoselective fashion. In their strategy, bicyclic ether 61 was found to be an important intermediate, since it contains the iridoid scaffold as well as bearing a carbonyl functionality at the C6 position and tosylate at the C4 position. The manipulation of the C6 carbonyl moiety in 61 makes the construction of cyclopentane ring of the desired natural products (65, 68, 68′, 70, 71, 72, 75, 75′, 79) possible whereas the tosylate present at C4 can be exploited for the construction of tetrahydropyran ring, which can be extended to the δ-lactone ring or easily discarded in the framework of scholarein A 80. In addition, total synthesis of some other natural products can be simplified by having easy access to an iridoid. For example, the total synthesis of 7-*epi*-boschnialactone 68′.

**Scheme 10:**
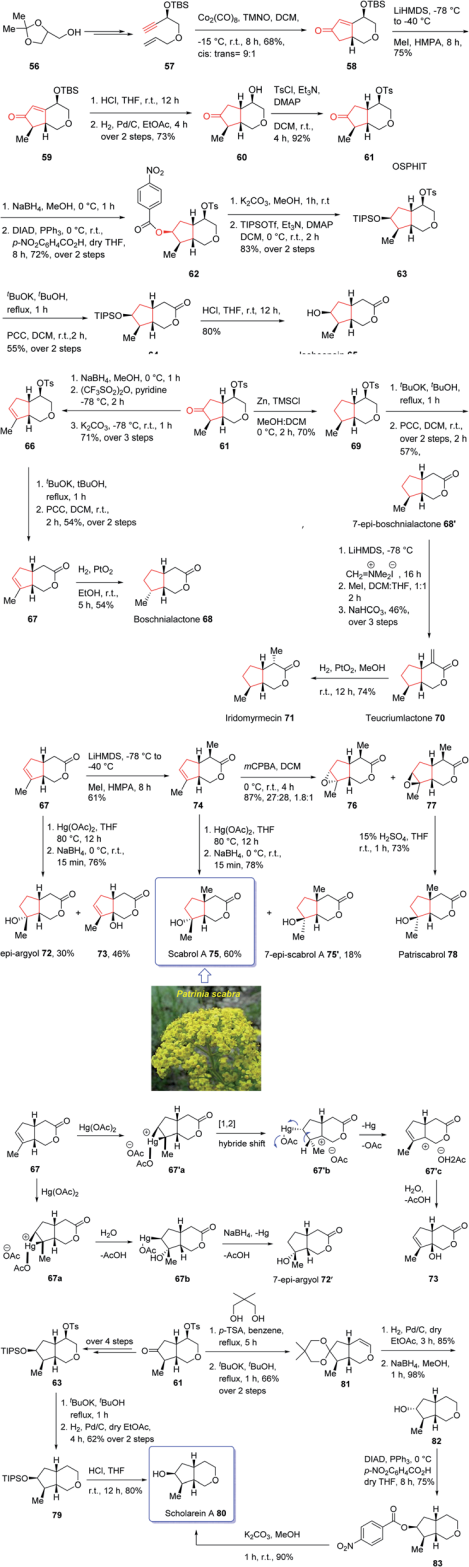
Total synthesis of noriridoid scholarein A 80.

This strategy is started with the easily accessible glycerol acetonide 56,^88,95^ which in two steps was converted into the enyne 57 as an appropriate precursor of PKR. The latter in crucial step was subjected to diastereoselective conventional IPKR^88^ in the presence of Co_2_(CO)_8_, TMNO to afford compound 58 which was then transformed into the important intermediate 61 in three steps. This important intermediate 61, was initially treated with Zn/TMSCl to undergo Clemensen reduction^96^ to give compound 69 which after two steps involving manipulation of its C4-tosylate moiety, 69 was converted into the δ-lactone affording the C7-epimer of boschnialactone 68′ in good yield, with identical spectroscopic data to that previously reported.^97^ On the other hand, intermediate 61 was converted into TIPS protected bicyclic lactone 64. TIPS deprotection in compound 64 in the presence of 10% HCl in THF gave the desired natural product iridolactone isoboonein 65 in satisfactory yield. The spectral data of this synthetic compound 65 were found being identical to those recorded for natural isoboonein.^93^ After successful synthesis of natural product 65, the synthesis of iridoids 71, 68 and 70 from the key intermediate 61 was contemplated. On the already prepared epimeric natural product 68′, an exocyclic methylene group was introduced at the C4 position to obtain the other desired natural product 70 and another natural product 71 was subsequently provided through the stereoselective exocyclic double bond reduction from the *exo* face. The spectral data of the synthetic compounds 71 and 70 were compared with those already recorded and reported for the natural products,^94,98^ and found being identical. Finally, noriridoid scholarein A 80 was synthesized from intermediate 61. The latter was first converted into intermediate 63 as a TIPS ether in several steps. Then, with 63 available in hand, its C4-tosylate group in the tetrahydropyran ring was eliminated and the resultant dihydropyran was oxidized to obtain the δ-lactone, the desired natural product isoboonein 65 (Scheme 10).^94^

Hamigeran A–D are members of a family of metabolites which are isolated from the extract of sponge *Hamigera tarangaensis* by Cambie and co-workers in 2000.^99^ They actually are a small class of brominated terpenes. Among members of this family, hamigeran-B 97 has exhibited remarkable biological potency as it showed 100% virus inhibitory property toward both herpes and polio viruses with negligible cytotoxicity.^100^ Its tricyclic structural backbone comprising an aromatic ring, has attracted the attention of organic synthetic chemists. Therefore, several research groups have focused on its total synthesis. The first asymmetric synthesis was achieved by Nicolaou and co-workers relied on an asymmetric Diels–Alder reaction as key step,^101^ Clive *et al.* employed radical cyclization for the construction of the five-membered ring conducting both a racemic and asymmetric synthesis.^102^ Thereafter, Trost and co-workers applied an asymmetric allylic alkylation as the source of asymmetry in a novel synthetic strategy for total synthesis of hamigeran-B.^103^ Wright *et al.* accomplished and reported the synthesis of hamigeran scaffold by employing an effective electro-oxidative coupling reaction.^104^ Very recently, Lovely and co-workers completed the framework of the tricyclic structure of hamigeran using Pauson–Khand reaction. In this strategy, cyclization only occurred when the olefin-containing group was tethered to the aromatic backbone to diminish its conformational movement. To this purpose, they selected silylene protecting group. Then, effective formation of the aryl enyne from a salicylic acid derivative was achieved through *ortho*-lithiation and Sonogashira cross-coupling reaction.

**Scheme 11:**
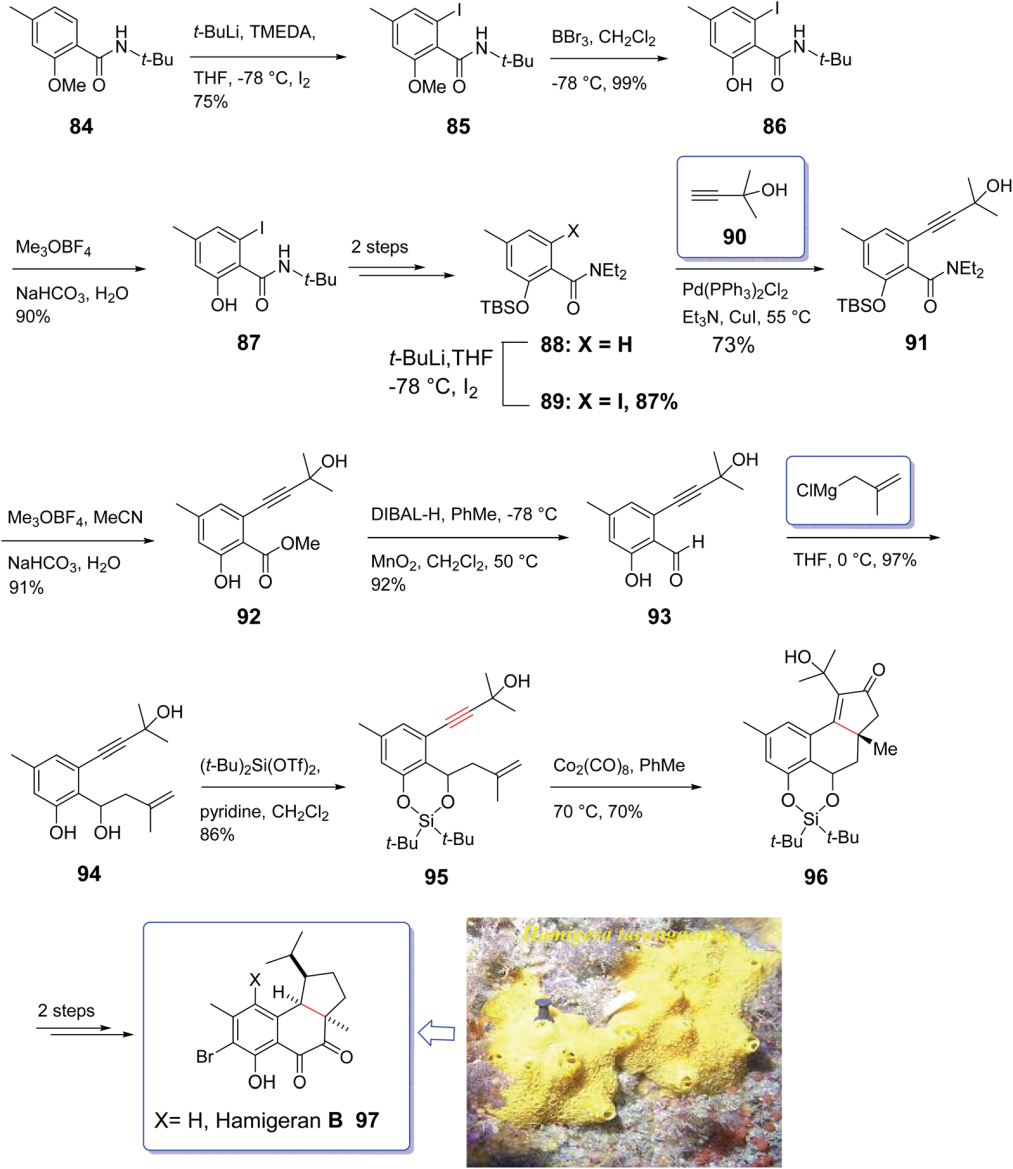
Total synthesis of hamigeran B 97.

A concise strategy for the total synthesis of hamigeran B 97 commenced from the easily accessible *tert*-butyl amide 84 (ref. 105) which was provided by standard acylation of salicylic acid. The latter upon treatment with *n*-BuLi leading to the formation of aryl lithium which was trapped with iodine to give 85. Upon cleavage of the methyl ether in 85 with excess BBr_3_, amide 86 was obtained upon treatment with Meerwein's salt and then aqueous base. The methyl ester was provided in 89% yield *via* three sequential steps. Compound 87 was then converted into 88 in two steps. The latter was then subjected to Sonogashira cross-coupling with 2-methyl-2-butynol which upon treatment with 90, hydrolysis of the amide employing the two-step reaction *via* the imitate gave 91. Then, compound 91 underwent desilylation to afford 92. Oxidation state adjustment of 92*via* reduction using DIBAL-H gave benzyl alcohol and oxidation with MnO_2_ afforded the corresponding aldehyde 93 as key intermediate. Reaction of the latter with methallylmagnesium chloride proceeded cleanly to give the diol 94, which upon treatment with (*t*-Bu)_2_Si(OTf)_2_ afforded the silylene derivative 95 in high yield. Pleasantly, the enyne 95 was converted into the Co_2_(CO)_8_ complex, with subsequent thermal activation at 70 °C in toluene under PKR resulted in the construction of desired tetracyclic adduct 96 in 70% yield as a sole diastereomer.^106^ The desired stereochemical biases was induced by intramolecular PKR resulting in the placement of the peripheral *exo* substituents.^107^ Finally, the latter was converted to the desired natural product hamigeran B 97 in two steps (Scheme 11).^104^

### 2.2. Terpenoids

**Scheme 12:**
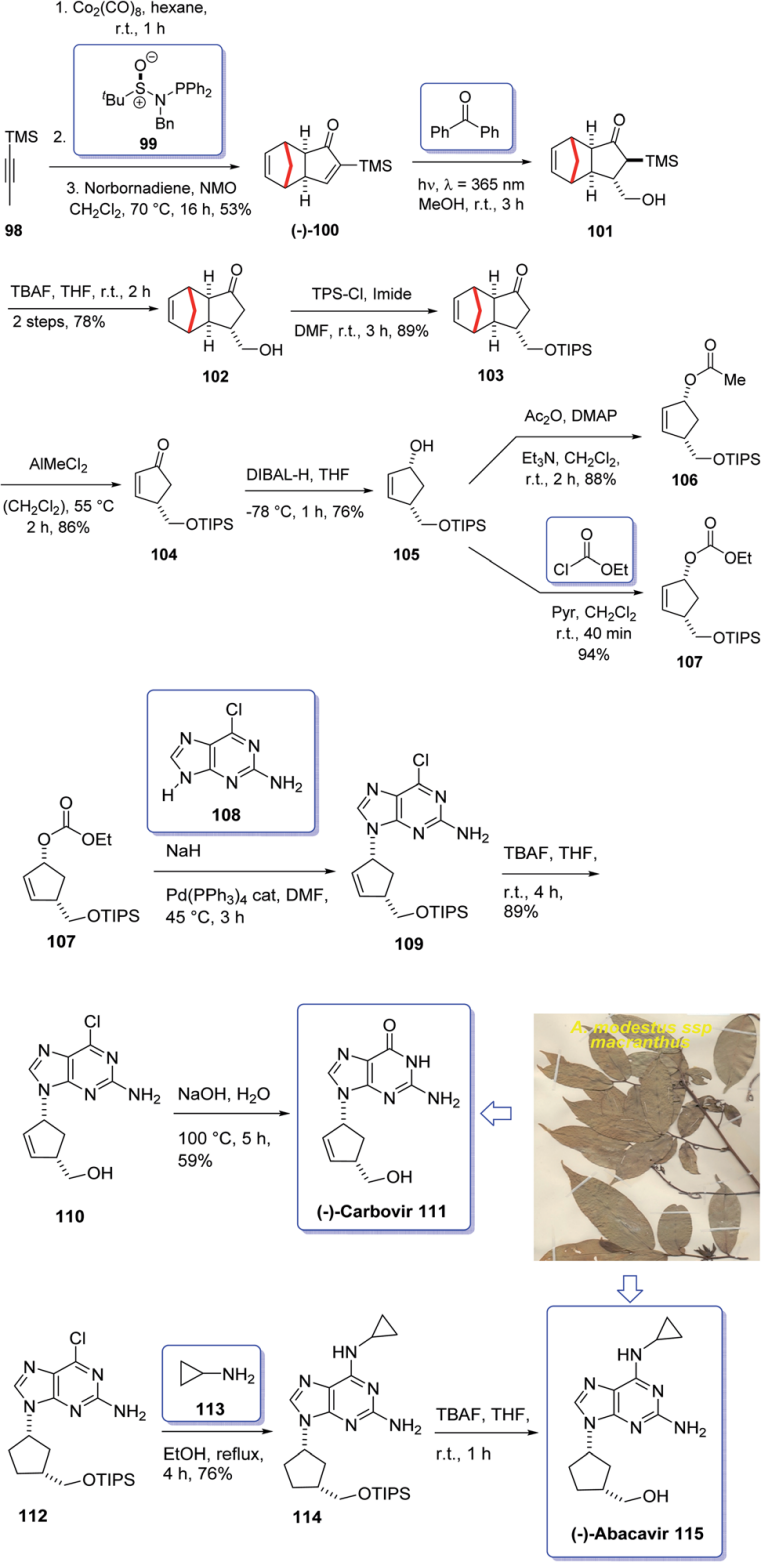
Total synthesis of (−)-carbovir 111, (−)-abacavir 115.

Terpenoids represent a highly diverse group of natural products with wide applications. Terpenoids, also known as isoprenoids, are the most numerous and structurally diverse natural products found in many plants. Several studies, *in vitro*, preclinical, and clinical have confirmed that this class of compounds displays a wide range of very important pharmacological properties. About 60% of known natural products are terpenoids. The diverse collection of terpenoid structures and functions have provoked increased interest in their commercial use resulting in some medical applications being registered as drugs on the market. Nowadays, nucleoside analogues have attracted the interest of synthetic organic chemists due to their significant biological activities and have been useful as antiviral and antitumor drugs.^108^ A novel meroterpenoid named artabotramide was obtained from the petroleum ether extract of the root barks of *Artabotrys modestus* subsp. *macranthus* Verdc by Baggio in 1978.^109,110^ Furthermore, the 2-azabicyclo [2.2.1]-hept-5-en-3-one (ABH) moiety present in artabotramide is of medicinal potential and one of the target pharmacophores in the synthesis of anti-retroviral carbocyclic nucleoside analogues such as (±)-carbovir 111 and abacavir 115.^110–113^ Thus, the unprecedented isolation of artabotramide from *A. modestus* ssp *macranthus* suggests the plant species to be a potential bio-resource for further investigation of carbocycles that are potentially important in biomedical research. Among them, AZT (Zidovudine), antiviral toward HIV, and Acyclovir (Zovirax), antiviral toward Herpex simplex, are well-known prescribed market purchasable drugs.^114^ Carbanucleosides establish a remarkable class of nucleoside analogues.^115^ Aristeromycin and neplanocin are natural product carbocyclic nucleosides showing antitumor and antiviral activity as well as exhibiting better metabolic stability to phosphorylases comparing with their glycosidic relatives.^116^ Carbovir 111 and abacavir (Ziagen) 115 are actually synthetic five-membered ring carbanucleosides. Since carbovir showed toxicity, it was not developed beyond the preclinical stage but abacavir was approved synthesized and launched for the treatment of HIV. Compounds 111 and 115 have been prepared by various pathways, initially *via* enzymatic resolution, kinetic resolution, as well as asymmetric synthesis stating from sugars.^117,118^ Nevertheless, up to date establishment of a general strategy *via* stereoselective synthesis have been largely overlooked.^119^ However, in 2005, Schmalz *et al.*^120^ achieved and reported a novel strategy for the total synthesis of carbocyclic nucleosides 111 and 115 including an intramolecular Pauson–Khand reaction^121^ as the key step. In this strategy, execution of kinetic resolution with the Corey's CBS reagent is required to obtain enantiopure compounds. Recently, several practical enantioselective versions of the intermolecular Pauson–Khand reaction have been reported^122^ which resulted in the formation of cycloadduct in high yield and optical purity. Armed with this finding, it was envisaged compound 99 could be an appropriate starting material for the total synthesis of many carbanucleosides. Based on the above reaction, an approach for asymmetric synthesis of (−)-carbovir 111 and (−)-abacavir 115 depended on asymmetric intermolecular PK reactions were designed for the total synthesis of carbanucleosides. This approach was designed based on readily accessible cyclopentenone 100 which is provided by the reaction of trimethylsilylacetylene 98 with norbornadiene *via* asymmetric PKR and retro-Diels–Alder reaction to give (−)-100 (Scheme 12).^122^ Thus, the vital step of this synthetic strategy is the stereoselective introduction of ad^1^-synthon into cyclopentenone 100*via* intermolecular PKR.^123^

In this way, both racemic and optically active PK adduct 104 were prepared at multigram quantities with ee is of >99%. Then, compound 103 in excellent yield and with complete stereospecificity was obtained by irradiating a solution of 100 in methanol at 365 nm in the presence of benzophenone as a triplet sensitizer, following a method developed by Fraser-Reid *et al.*^124^ Next, the TMS group of 103 was readily deprotected using TBA to give the required intermediate alcohol 102 in 78% yield. Thereafter, the hydroxyl group of 101 was protected as triisopropylsilyl ether under standard conditions to afford 103 in 89% yield. The protected hydroxyethyl cyclopentanone 103 was then subjected to the retro-Diels–Alder conditions reported by Grieco^125^ using AlMeCl_2_ as a Lewis acid and maleic anhydride as a cyclopentadiene scavenger to afford cyclopentenone 104 in 86% yield. At this stage, the latter was reduced using DIBAL-H at low temperature to give allyl alcohol 105 in satisfactory yield. Upon allylic substitution, compound 105 was readily derivatized to the corresponding acetate 107 and carbonate 106. Gratifyingly, reaction of carbonate 106 using sodium hydride as a base afforded the key nucleoside in 84% yield with a 4 : 1 dr of N9/N7 regioisomers. The desired regioisomer at N9 109 was obtained in 67% yield after chromatographic purification, and ee > 99% determined by chiral HPLC. Finally, the key intermediate 110 was converted into desired natural products *i.e.* enantiomerically pure (−)-carbovir 111 and (−)-abacavir 115 using compound 110 and 114, respectively (Scheme 12).^115^

**Scheme 13:**
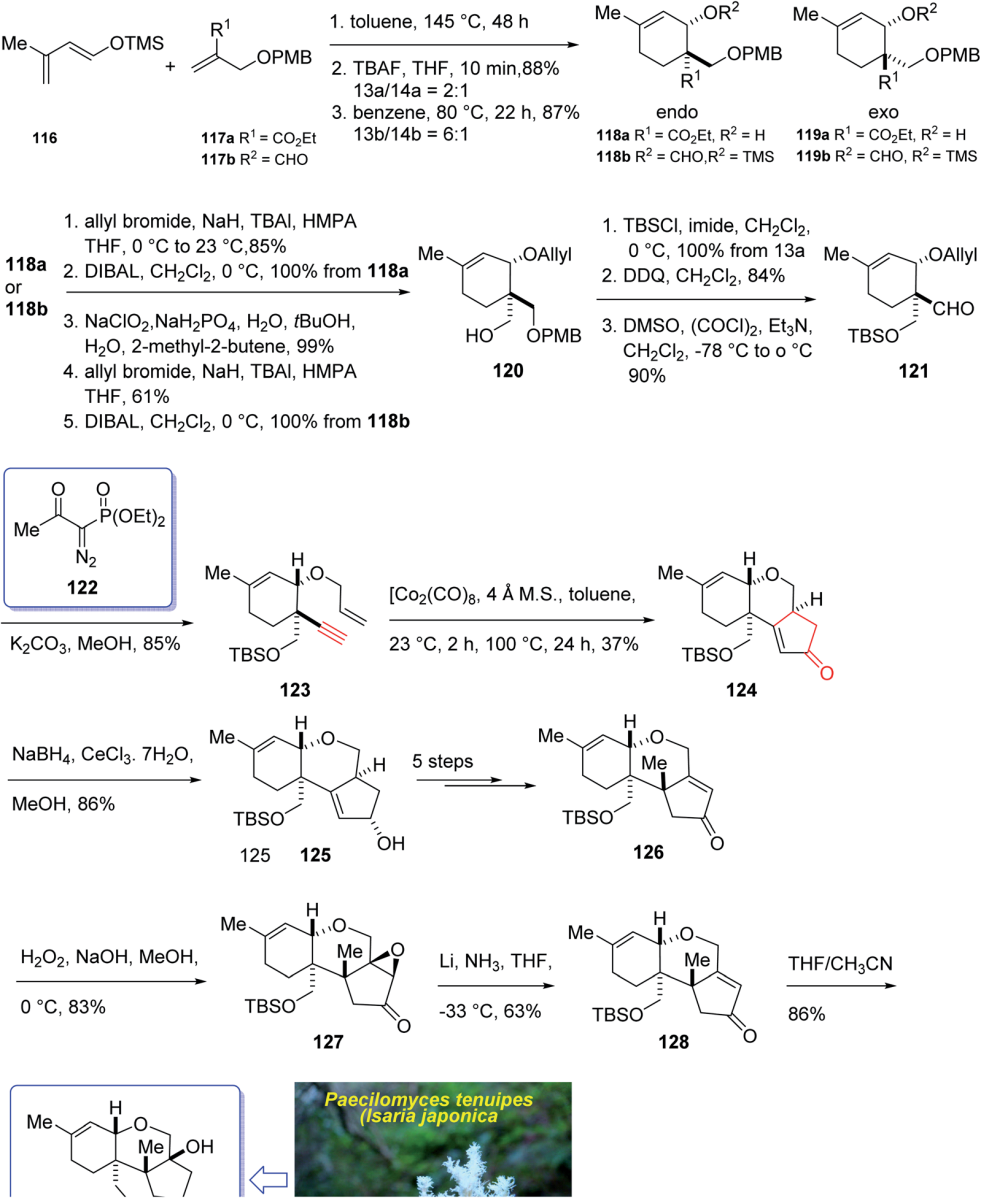
Total synthesis of paecilomycine A 129.

In 2004, Oshima *et al.*^126^ isolated several paecilomyces tenuipes terpenoids from extract of cultured fruiting bodies of *Paecilomyces tenuipes* (*Isaria japonica*). It was actually a common entomopathogenic fungus employed as traditional remedy and healthy foods in China.^126^ Among them, compound 129 (paecilomycine A), at 10 nm, is able to promote neurite outgrowth in PC 12 cells. It was also known that paecilomycine A 129 is considerably more potent than scabronine G in increasing NGF levels. In 2007, the appearance of a fascinating skeleton for the total synthesis of paecilomycine A 129 (ref. 127) was stimulated by Danishefsky and co-workers for the isolation and structure elucidation. In 1961, Martin and Hill reported an initial total synthesis of racemic 129*via* Diels–Alder reaction to many early hindrances in their efforts^128^ as well as using Pauson–Khand reaction. Initially, the Diels–Alder reaction of 116 (ref. 129) and 117a,^130^ under severely thermal conditions (at approximately 145 °C in toluene) followed by deprotection, afforded a 2 : 1 mixture of 118a and 119a with 88% yield. As illustrated, *O*-alkylation performed to give the corresponding allyl ether. Reduction of the ester group produced compound 120 which followed by protection as a silyl ether and deprotection of the PMB group followed by oxidation of the obtained alcohol to aldehyde afforded compound 121. The compound 123 was afforded *via* Bestmann–Ohira reagent (dimethyl 1-diazo-2-oxopropyl phosphonate) with compound 121. An important compound available in hand 123, was submitted to intramolecular Pauson–Khand reaction at 100 °C (ref. 19 and 131) to afford the sole stereoisomer 124 in 37% yield. The compound 126 was transformed to desired natural product paecilomycine A 129, involving several group functional transformation as illustrated in (Scheme 13).^127^

In 2008, Sun *et al.*^132^ for the first time isolated propindilactone G 152 (ref. 132) which is a member of a novel family of nor-triterpenoids^133^ from different species of Schisandraceae family (*Schisandra propinqua* var. *propinqua*) from Southeast Asia.^132^ It has been used as folk herb in China for liver protection and immune-regulation for a long time.^133^ Structurally, propindilactone G 152 contains an exceptional 5/5/7/6/5 pentacyclic scaffold having seven chiral centers which three of them are quaternary stereogenic centers.^134^ Primary biological screening of propindilactone G 152 specified that these kinds of nortriterpenoids show auspicious anti-HIV potency.^135^ Their, interesting chemical structures in combination with their insufficiency in nature, which restricts their further biological screening, have prompted great interest among^136^ synthetic organic chemists to design a pathway for the total synthesis of propindilactone G1.

In 2015, a brief total synthesis of (+)-propindilactone G 152 using Pauson–Khand reaction as a key step was accomplished and reported by Yang *et al.*^137^

**Scheme 14:**
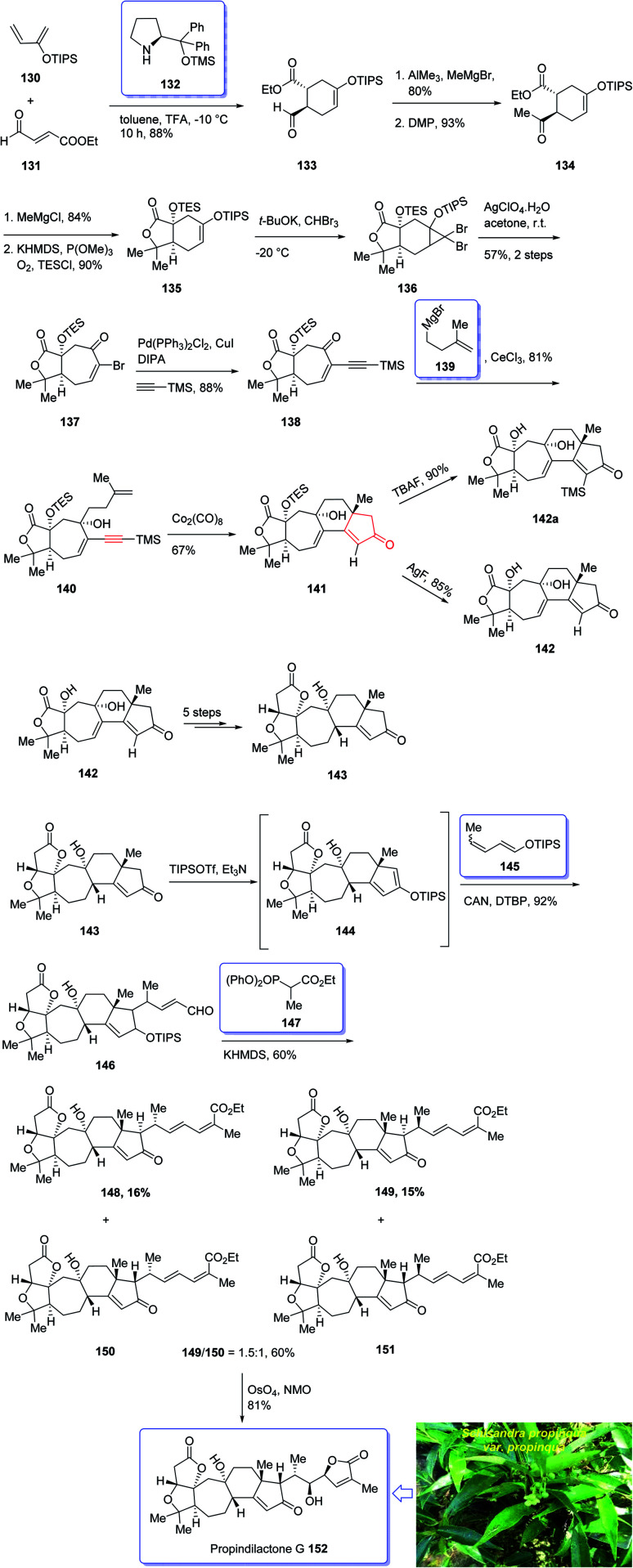
Total synthesis of (+)-propindilactone G 152.

This strategy started with an asymmetric Diels–Alder reaction of diene 130 and dienophile 131 in the presence of a chiral ligand (Jorgensen–Hayashi catalyst) 132 which afforded (−)-ester 133 in high chemical yield and in excellent ee (98%). After several steps, the latter was transformed into enyne 140 as a sole isomer. Then, the latter was subjected to PKR conditions which upon treatment with Co_2_(CO)_8_ in the presence of Celite^138^ in refluxing toluene afforded cyclopentenone subunit 141 bearing an all-carbon quaternary chiral centers. After several steps, the latter was converted to a mixture of 148, 149 and 150/151. Pleasantly, compound 150 was converted into the desired natural product propindilactone G 152 upon treatment with OsO_4_ as oxidant and in the presence of NMO^139^ as a co-oxidant. These reactions desired the total synthesis of (+)-propindilactone G 152 in only twenty steps (Scheme 14).^166^

**Scheme 15:**
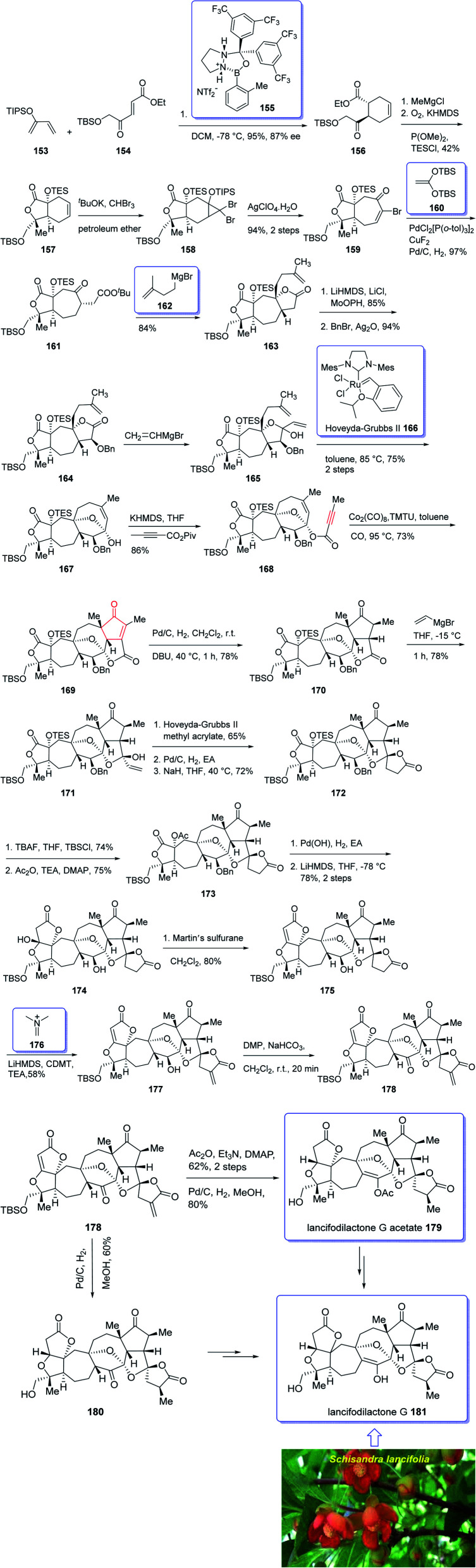
Total synthesis of lancifodilactone G 181.

In 2005, Sun and his research group initially isolated lancifodilactone G 181, as one of the most important members of the schinortriterpenoids family, from the extract of *Schisandra lancifolia*^140^ which had been used as anti-hepatitis, antitumor, and anti-HIV agents as traditional medication.^133^ Due to these biological activities and interesting chemical structure of 181, many attempts were made to their total synthesis,^141^ with the goal of fast-tracking of the assessment of its pharmacological activity. Asymmetric total synthesis of structurally fascinating and highly oxygenated lancifodilactone acetate G 7 was accomplished in twenty-eight steps from commercially available 2-(triisopropylsiloxy)-1,3-butadiene 153 reported by Yang *et al.* in 2017.^142^ The total synthesis started with asymmetric intermolecular Diels–Alder reaction^143^ of diene 153 with dienophile 154 catalyzed by oxazaborolidine 155 (ref. 144) to provide ketoester 156 in satisfactory chemical yield and 87% ee. After several steps, the latter was transformed into enyne 168 as an appropriate PKR precursor. The enyne 168 was then subjected a PKR upon treatment with the complex of tetramethyl thiourea (TMTU) and Co_2_(CO)_8_ under already secured optimal conditions to afford enone 169 in 73% yield as a single isomer. Next, the enone 169 was transformed after several steps into ketone 178. The latter can be converted to lancifodilactone G acetate 179 in two steps including a Pd/C-catalyzed hydrogenation. On the other hand, ketone 178 was converted into 180 which after several steps was converted to the desired natural product lancifodilactone G 181 (Scheme 15).^142^

(±)-Schindilactone A 210 is a member of family of Schisandraceae which is valuable from both economic and medicinal points of view.^145^ More than 20 species of Schisandraceae were found in China which have extensively been used as traditional medicines^146^ over 2000 years in 2008 Han-Dong Sun research group isolated over 70 nortriterpenoids from Schisandraceae.^147,148^ Among them, schindilactone A 210 (ref. 149 and 150) was found being conspicuous member with eminent biological potencies including inhibition of tumor growing and hepatitis, and also as anti-HIV-1.^147^ In spite of its prominence, only small amounts of schindilactone A 210 can be obtained from natural sources even for its biological potencies screening, thus, its total synthesis has attracted much attention of synthetic organic chemists.

**Scheme 16:**
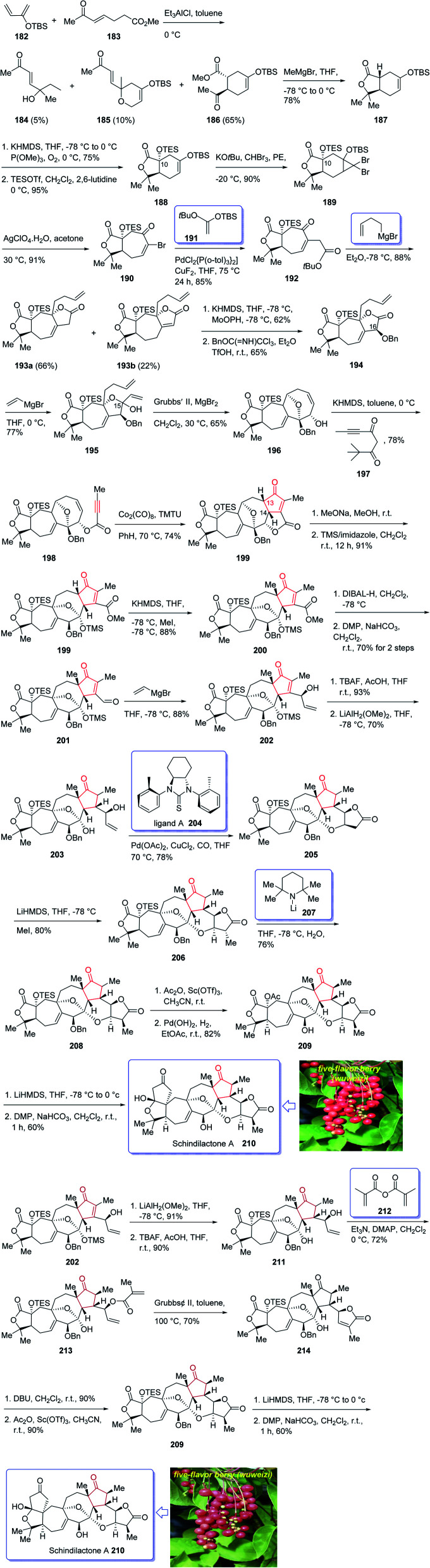
Total synthesis of (±)-schindilactone A 210.

In 2012, Yang *et al.*^145^ accomplished and reported the total synthesis of schindilactone A 210. Their strategy involved (a) an Ag-mediated ring-expansion reaction to obtain vinyl bromide 180 from dibromocyclopropane 189; (b) a Pd-catalyzed cross-coupling of vinyl bromide 190 with a copper enolate to provide ketoester 192; (c) a RCM reaction to obtain oxabicyclononenol 196 from diene 195; (d) construction of cyclopentenone segment in substrate 199*via* catalyzed Pauson–Khand reaction. This total synthesis started with hetero-Diels–Alder reaction between diene 182 and dienophile 183 in toluene at 0 °C in the presence of Et_2_AlCl^151^ to furnish a mixture containing compound 184 (about 5% obtained from the alkylation of Et_2_AlCl to the keto group in dienophile), compounds 185 and 186 (about 10%). Then, upon the treatment of ester 186 with MeMgBr, lactone 187 was obtained in good yield. The latter was converted to enyne 198, as an appropriate PKR precursor, after several steps involving various functional group transformations. In a key step, enyne 198 was subjected to PKR under the secured optimal PKR conditions (Co_2_(CO)_8_/TMTU in dry benzene under a CO atmosphere (balloon) at 70 °C for 4 hours) affording compound 199 in satisfactory yield. Then, the latter converted into alcohol 209 after several steps. Next, alcohol 209 was subjected to a Dieckmann-type condensation, upon treatment with LiHMDS in THF at −78 °C, followed by oxidation using DMP to afford the final desired natural product schindilactone A 210 in 60% overall yield over two steps (Scheme 16).^145^

### 2.3. Diterpenes and diterpenoids

**Scheme 17:**
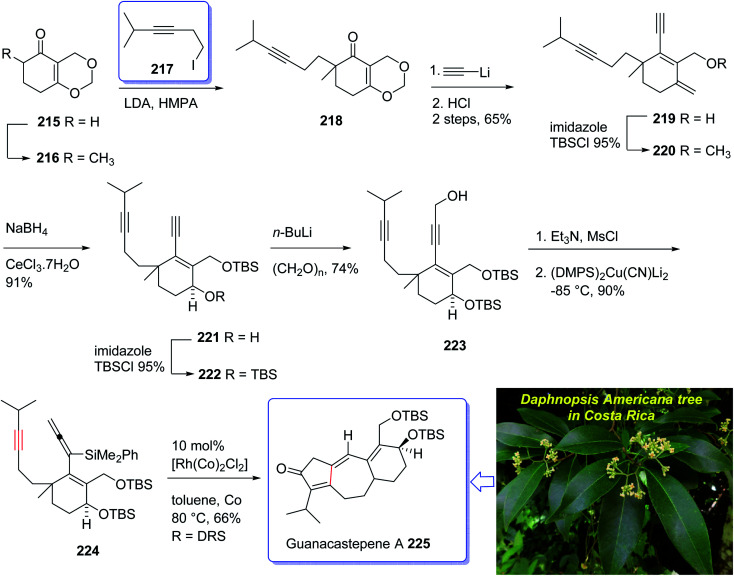
Total synthesis of guanacastepene A 225.

The active component isolated from the extracts of the branch of a *Daphnopsis americana* tree in Costa Rica was so-called guanacastepene A. It is actually a diterpene having a unique carbon skeleton structure.^152^ It exhibited very high potency towards faecalis (VREF) pathogens, methicillin-resistant *S. aureas* (MRSA) and vancomycin-resistant E.^152^ Guanacastepene A 225 has a unique carbon skeleton and a highly functionalized upper half, thus it has attracted much attention of synthetic organic chemists worldwide, as an interesting compound for total synthesis. Due to these waves of interest, the total synthesis of 225 has been attempted and successfully achieved by several research groups.^153^ Danishefsky *et al.*^154,155^ accomplished and reported the first total synthesis of guanacastepene A 225, but before long thereafter Snider *et al.* achieved and reported total synthesis of 225.^156^ Interestingly, both of these protocols started with formation of the five-membered ring with subsequent annulations of the seven- and six-membered rings. A conceptually new pathway to the highly functionalized tricyclic core of guanacastepene A 225 was designed through the use of rhodium(i)-catalyzed intramolecular allenic Pauson–Khand reaction for the construction of seven-membered rings.^157^ This strategy commenced with Smith's enone 215 (ref. 158) which in enolate form was alkylated with an appropriate alkyne iodide in the presence of LDA to afford compound 218 in moderate yield.^159^ Reaction of the enone 218 with lithium acetylide ethylenediamine complex *via* sequential hydrolysis of the crude product with HCl gave enone 219 in 65% yield over two steps. The hydroxyl group of the latter was protected using TBSCl to afford the corresponding silyl ether 220 in 95% yield. The carbonyl group of the latter, upon reduction following the Luche protocol provided the corresponding secondary alcohol 221 as a mixture of two diastereomers (3 : 1 ratio). Protection of the allylic alcohol gave *tert*-butyldimethylsilyl ether 222 in 88% yield over the two steps. Deprotonation of the terminal alkyne using *n*-BuLi at −78 °C, followed by the addition of paraformaldehyde afforded the corresponding propargylic alcohol 223 in satisfactory yield. The alcohol 223 upon treatment with Et_3_N and MsCl produced a mesylate, which without purification was added directly to (Me_2_PhSi)_2_Cu(CN)Li_2_ at −85 °C to provide the 3,3-disubstituted allene 224 in 90% yield over two steps. Having pure allenyne 224 available in hand, it was treated *via* Pauson–Khand reaction conditions (10% mol [Rh(CO)_2_Cl]_2_ in toluene at 80 °C) to give the 4-alkylidene cyclopentenone 225 as a single product in 65% yield which in fact is the tricyclic ring core system of natural product, guanacastepene A 225 (Scheme 17).^160^

Ingenol 233 is a highly oxygenated tetracyclic diterpene which in racemic form imitates the task of diacylglycerol, being the endogenous activator of protein kinase C.^161^ It is actually isolated from Euphorbia family and its structure was unambiguously elucidated and reported in 1968 by Hecker *et al.*^162^ This natural product 233 shows weak activity against protein kinase C.^163^ Ingenol 233 which is the parent compound of several dozen of natural product ingenanes, has the same carbon scaffold but diverse peripheral functionalities.^164^ Besides their fascinating “inside-outside” bridged BC ring system,^165^ the ingenanes show diverse biological activities.^166^ In 1980s, Winkler *et al.* inspired by this significant biological function and complex architecture attempted the total synthesis of 233.^167^ In 1997, the same group used the intramolecular dioxenone photocycloaddition to create its exceptional stereochemical feature.^161^ In 2002, Winkler *et al.*^167^ accomplished and reported the first total synthesis of racemic ingenol. The total synthesis of 233 was completed in forty-two steps in overall yield of 0.042% from commercially available.

**Scheme 18:**
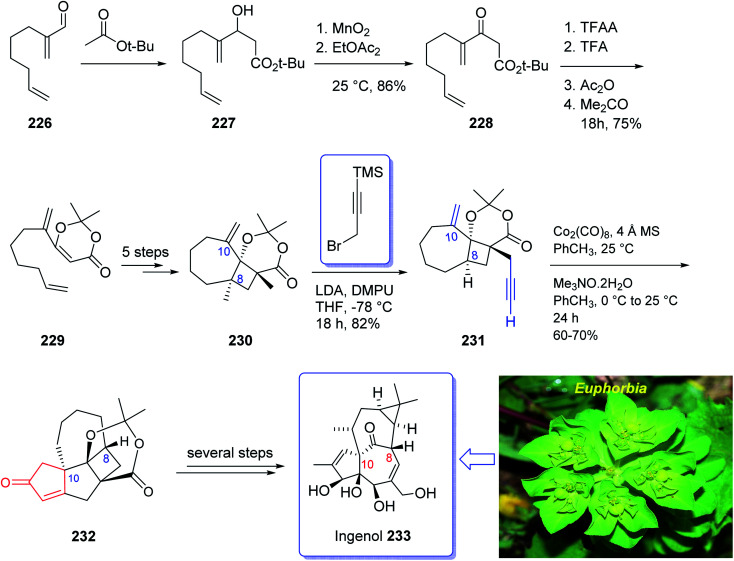
Total synthesis of ingenol 233.

In 2005, Winkler *et al.*^168^ achieved and reported the total synthesis of racemic ingenol 233 beginning from unsaturated aldehyde 2-methyleneoct-7-enal 226 which was synthesized in a one-pot fashion involving Swern oxidation of 7-octen-1-ol followed by reaction of the intermediate aldehyde with Eschenmoser's salt.^169^ Compound 226 then reacted with the conjugate base of *tert*-butyl acetate to afford compound 227, which upon oxidation by MnO_2_ gave ketoester 228. Treatment of 228 under dioxenone-forming conditions (TFAA, TFA, Ac_2_O, Me_2_CO) resulted in the formation of the dioxenone photosubstrate 229 in satisfactory yield. After five steps, the desired methylene photoadduct 230 was obtained. Alkylation of the conjugate base of 230 (LDA, THF, DMPU, −78 °C) with 3-trimethylsilylpropargyl bromide followed by desilylation with TBAF (THF, 100%) the Pauson–Khand substrate 231 was provided. It is worthwhile to mention that the Pauson–Khand reaction of 231 in the presence of the Me_3_N-*N*-oxide dehydrate was substantially more effective than the reaction employing anhydrous Me_3_N-*N*-oxide. After several steps involving various functional group transformations, compound 232 was converted to the desired natural product ingenol 233. In summary, the target 233 was obtained from 226 in overall yield of 0.042% (Scheme 18).^168^

The aquariolides are actually classified as cyclic diterpenes. They were initially isolated from *Erythropodium caribaeorum*, but in 2002 aquariolide A 244 was identified from cultured specimens of this gorgonian^170^ by the Andersen research group. Andersen and co-workers in 2003 achieved and reported the isolation of aquariolides A, B, and C from animals growing in the wild.^171^ In fact, the distinctive feature of these naturally occurring compounds is the “aquariane” backbone, which contains two five-membered rings fused to a nine-membered ring *E. caribaeorum* was found being a source of briarane ditepenes,^172^ and the aquariolides which are supposed to generate biosynthetically from a briarane precursor through a di-π-methane rearrangement followed by vinyl-cyclopropane rearrangement.^171^ A brief biological screening disclosed that aquariolides B and C showed moderate *in vitro* cytotoxicity against human breast cancer MCF-7 cells.^202^

In 2006, the total synthesis of 244, as a prelude was accomplished and reported by Burnell and co-workers involving a diastereoselective Pauson–Khand reaction and subsequent ring expansion.^173^

In this approach, the total synthesis of aquariolide A 244 is commenced from benzyl ester 234, which was converted into ynone 235 in 85% yield *via* Yamaguchi procedure.^174^ Then, ketone 235 was subjected into geminal acylation upon reaction with 236 in the presence of BF_3_·OEt_2_ to provide diketone 237. Protection of the latter hence provided the extra advantage of further directing addition of a vinyl Grignard reagent *via* blocking the alkenyl face of the cyclopentanone. Thus, the mono reduction of diketone 237 using lithium tri(*tert*-butoxy)-aluminohydride afforded diastereomeric alcohols (238 and 239) in a ratio of 4 : 1. Keto-alcohol 239, the other product from the reduction of 237, was reacted with triethylsilane in TFA, and then protected by TBSCl and reacted with vinylmagnesium bromide in the presence of anhydrous CeCl_3_,^175^ followed by basic methanolysis of the TMS group to give compound 7 as the sole product. It was obtained upon treatment of the latter with Co_2_(CO)_8_ and trimethylamine *N*-oxide, under the Pauson–Khand reaction conditions (in dichloromethane at 30 °C) as a 4 : 1 ratio of inseparable diastereomers 241 and 242 (in a ratio of about 4 : 1 determined by NMR).

**Scheme 19:**
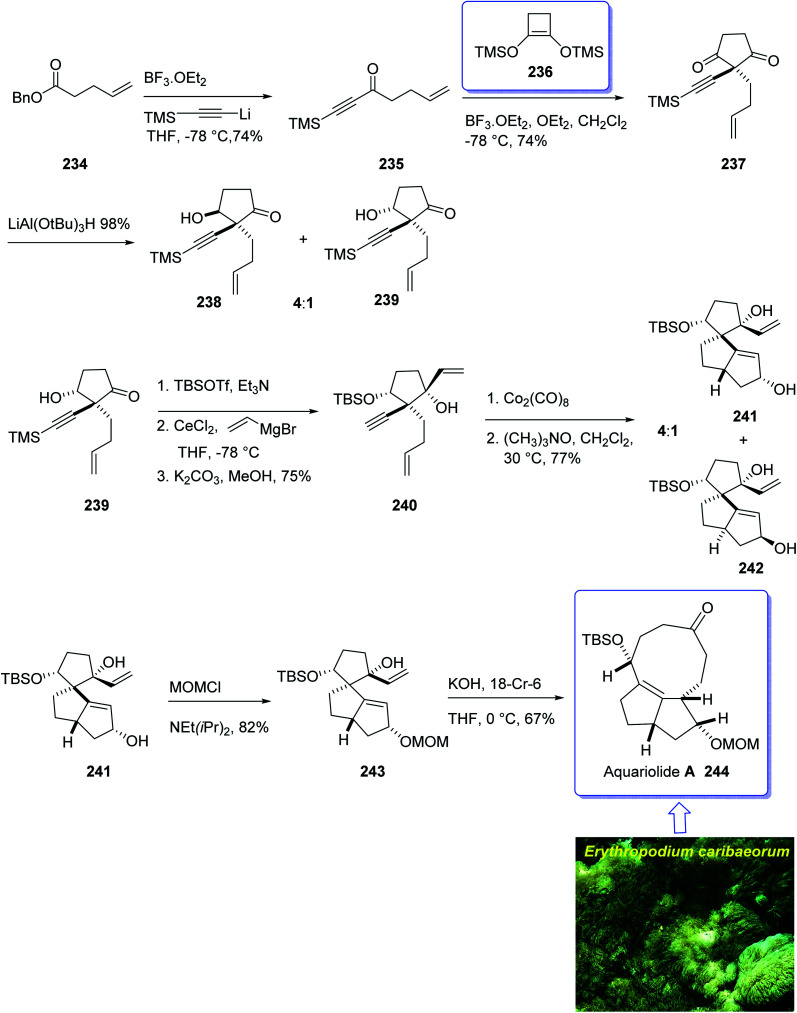
Total synthesis of aquariolide A 244.

Protection of the major alcohol 241 as a MOM ether afforded 243. As expected, subjecting 243 to appropriately basic conditions smoothly provided ketone 244. The latter *via* viable routes to the ring system of the aquariolide diterpenes have been accomplished in two steps *via* generation of 241 and 243 in which the latter was transformed to desired natural product aquariolide A 244 in 67% overall yield (Scheme 19).^173^

Cyanthiwigins^176^257 contain a cyclohepta[*e*]indene core and structurally belongs to the diverse cyathane class of diterpene.^177^ They were isolated from the extract of the marine sponges *Epipolasis reiswigi* and *Mermekioderma styx*. Biologically, cyanthiwigins were found being cytotoxic towards A549 lung cancer cells and primary tumor cells.^178^ Cyanthiwigins have 5-6-7 tricarbocyclic core with carbons in different oxidation states. An efficient total synthesis of cyanthiwigins 257 was achieved and reported by Phillips *et al.* in 2005.^179^ Then, the Stoltz research group developed and reported a brief strategy involving double asymmetric catalyzed alkylation and an RCM reaction as the key steps in the total synthesis of cyanthiwigins B, F, and G.^180^ In 2013, Gao *et al.* accomplished a brief synthetic strategy for the total synthesis of cyanthiwigins A, C, G, and H^181^ comprising a common intermolecular [4 + 2] cycloaddition and RCM reaction as vital steps.

**Scheme 20:**
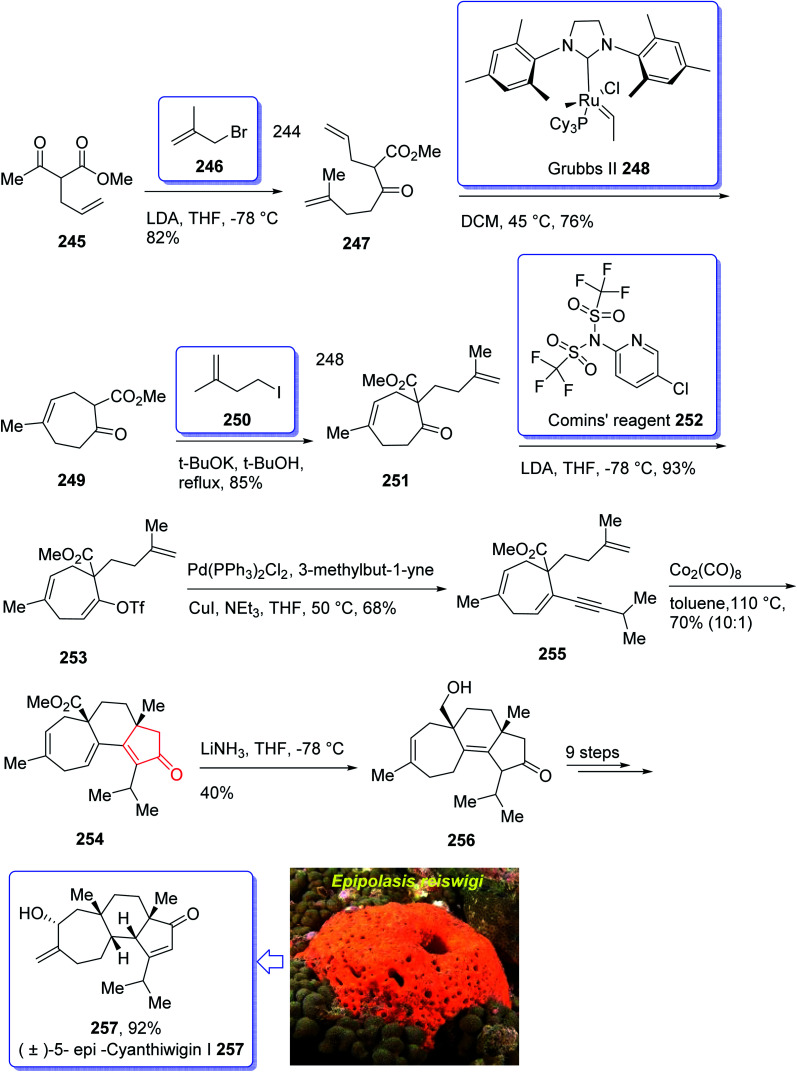
Total synthesis of (±)-5-*epi*-cyanthiwigin 257.

In 2018, Yang *et al.* developed a strategy for the total synthesis of 5-*epi*-cyanthiwigin I 257 using Pauson–Khand reaction (PKR) as a vital step. The total synthesis of 257 began with 245 which was reacted with allyl bromide 246 in the presence of LDA to afford diene 247 in high yield.^182^ The latter was further treated with Grubbs II catalyst 248 (ref. 183) to provide ketoester 249, which upon alkylation with 4-iodo-2-methylbut-1-ene 250 in the presence of KOBu-*t* in *tert*-BuOH afforded ketone 251 in high yield. The key intermediate 254 was obtained by converting the ketone group of the latter upon treatment with LDA to the corresponding enolate, which was subsequently reacted with Comins' reagent 252 to provide the respective vinyl triflate 253 in excellent yield. The latter was reacted with 3-methylbut-1-yne under Sonogashira coupling reaction conditions with enyne 254 in good yield. Next, enyne 254 was treated with Co_2_(CO)_8_ (1.2 equiv.) in refluxing toluene under PKR conditions for the construction of the indene core 255 of cyanthiwigins in good yield. Upon treatment of 255 with Li/NH_3_ in THF at −78 °C, compound 256 was obtained in 40% yield which after nine steps was transformed to the desired C5-*epi*-cyanthiwigin I 257 in 92% yield. The developed chemistry enables the total synthesis of 5-*epi*-cyanthiwigin I 257 in seventeen steps and can be used for the total synthesis of other cyanthiwigins (Scheme 20).^177^

**Scheme 21:**
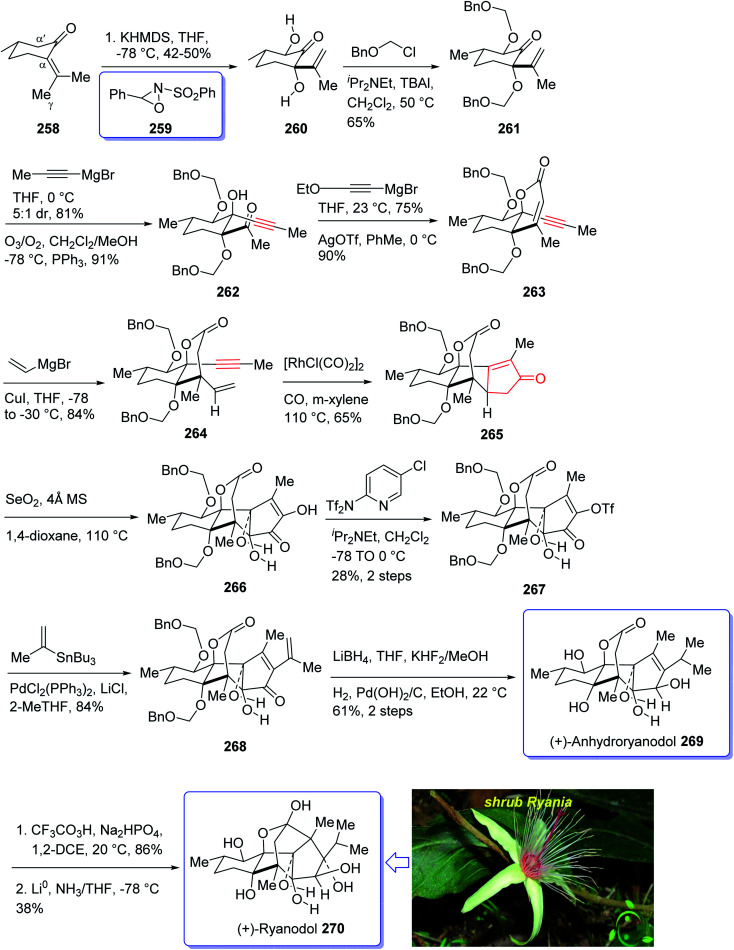
Total synthesis of (+)-ryanodol 270.

Naturally occurring compound, ryanodine^184,185^ and its hydrolysis product ryanodol 270,^185,186^ are among the most highly oxidized diterpenoids reported so far. Ryanodol 270 was isolated from the extracted tropical shrub *Ryania* speciose Vahl reported by Pepper and Carruth in 1945,^187^ and then by other research groups.^188^ It showed insecticidal properties^184^ and is an important family of ion channels that regulate intracellular Ca^2+^ release and play a key role in signal transduction.^189^ In 2016, Chuang *et al.*^190^ achieved and reported a brief total synthesis of (+)-ryanodol in fifteen steps stating from the commercially available terpene (*S*)-pulegone 258. In this strategy, the utilization of a Pauson–Khand reaction rapidly construct the carbon skeleton along with a SeO_2_-catalyzed oxidation to assemble three oxygen atoms *via* a single step. In this approach, reaction of (*S*)-pulegone 258 with KHMDS at −78 °C followed by dropwise addition of 259 gave α,α′-diol 260 which was isolated as a single diastereomer in 42% yield. Treatment of diol 260 with excess benzyl chloromethyl ether resulted in the protection of both alcohols as benzyloxymethyl ethers to afford 261. At this point, the D-ring was created by an effective four-step sequence. Initially, propynylmagnesium bromide was added to 261 at 0 °C which proceeded in 5 : 1 dr to give the equatorially disposed alkyne in 81% yield. The latter upon ozonolysis was cleaved to give methyl ketone 262. Then, the ketone was effectively transformed to α,β-unsaturated lactone 263*via* 1,2-addition of ethoxyethynylmagnesium bromide with subsequent sequential Ag-catalyzed cyclization and elimination reactions.^191^ Having lactone 263 available in hand, 1,4-addition of magnesium divinyl cuprate gave the respective enyne 264 as a sole diastereomer in satisfactory yield. In a crucial step, compound 264 was treated with 1 mol% [RhCl(CO)_2_]_2_ (ref. 30) under an atmosphere of carbon monoxide (PKR) gave the desired intermediate 265 as a sole diastereomer and in high chemical yield. This intermediate was transformed after four steps to afford (+)-anhydroryanodol 269 in 61% yield. The latter was then treated with trifluoroperacetic acid to give epianhydroryanodol epoxide which is subjected to reductive cyclization to give the desired natural product (+)-ryanodol 270 in 0.42% overall yield over fifteen steps starting from commercially available (*S*)-pulegone 258 (Scheme 21).^190^

Epoxydictymene 284 is a diterpene naturally occurring compound which is present in the brown alga *Dictyota dichotoma* isolated.^192^ In the early 1980s, Matsumoto *et al.*^193^ isolated cyclononane and hydroazulene diterpenoids and in 1983 Ishida *et al.*^194^ elucidated its structure as epoxydictymene 284. It contains a 5-8-5-5 (ref. 195) tetracyclic scaffold containing a strained *trans*-3-oxabicyclo [3.3.0] octane (*trans*-5-5).^196^

**Scheme 22:**
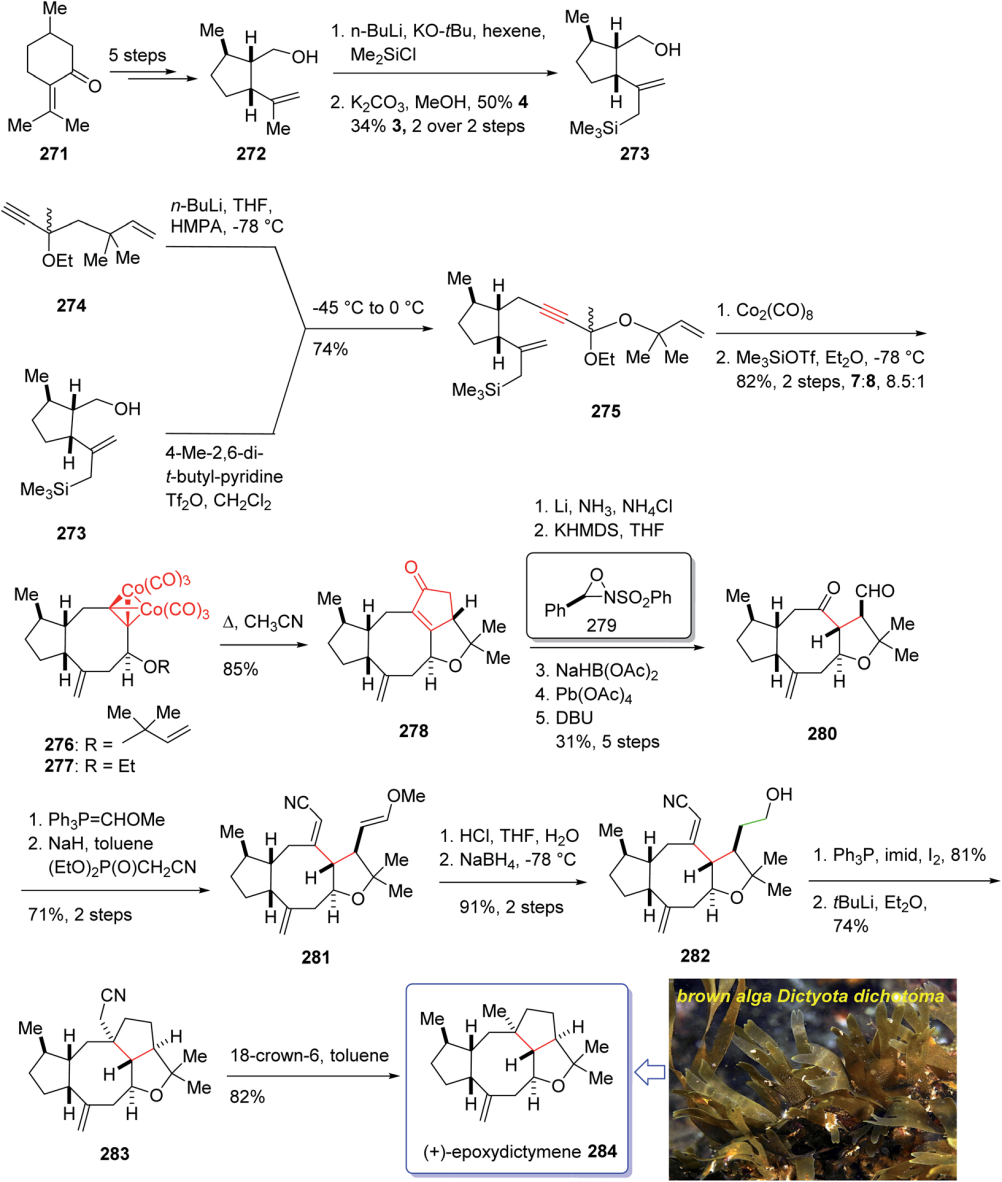
Total synthesis of epoxydictymene 284.

The total synthesis of 284 was achieved and reported by Schreiber and co-workers in 1994 (ref. 197) by employing Pauson–Khand reaction as a key step. The total synthesis started from commercially available (+)-puelgone 271 which after several steps was converted into compound 272. The latter was transformed in two steps involving protection of hydroxyl group by TMSCl in the presence of *n*-BuLi to afford compound 273. Reaction of lithium anion of 274 and alcohol 273 through displacement of a triflate ester under carefully controlled conditions, furnished alkyne 275 as suitable PKR precursor in high yield. In a crucial stage, the latter was treated with Co_2_(CO)_8_ under PKR conditions to afford the desired organometallic cluster, which was then subjected to catalyzed Lewis acid cyclization^197^ (Me_3_SiOTf, Et_2_O, −78 °C, 15 min) to furnish ethers 276 and 277 in 82% overall yield as an 8.5 : 1 mixture of diastereomerically pure compounds. After several steps, ether 276 was converted into nitrile 283 containing the complete framework and configurations of the target natural product. Reductive decyanation^198^ of nitrile 283 gave the desired natural product (+)-epoxydictymene 284 (Scheme 22).^192^

Plumisclerin A 298, an exceptional marine diterpenoid, was initially isolated by Reyes research group in 2010 from the samples of *Plumigorgia terminosclera* collected at Mayotte Island.^199^ Structurally, plumisclerin A 298 has a complicated and compact ring system bearing a fully-substituted cyclobutane (C ring), a bridged cyclohexane (D ring), a poly-substituted cyclopentane (B ring), as well as a fused dihydropyran ring (A ring). Its distinctive rigid tricyclo [4,3,1,0^1,5^] decane framework probably is the first reported of such a plumisclerane framework in the field of naturally occurring compounds. Dihydropyran ring (A ring) in plumisclerin A 298, is *trans*-fused to the cyclopentane ring (B ring), while, most terpenoids have a *cis*-fused dihydropyran ring.^200^ It also bears seven chiral centers involving two all-carbon quaternary chiral centers compactly spread in the molecule. Plumisclerin A 298 exhibits modest cytotoxicities towards numerous common tumor cells such as lung, colon cancer and breast cancers.^199^ The interesting structure and its valuable biological activity, make plumisclerin A 298 as an important target for synthetic organic chemists. An effective strategy for the synthesis of the tricyclo [4,3,1,0^1,5^] decane core (B/C/D rings) of plumisclerin A 295 was designed and successfully accomplished by Yao and co-workers in 2015.^201^ In this strategy, the Pauson–Khand reaction and a SmI_2_-catalyzed radical 1,4-conjugate addition play vital roles in the construction of fully functionalized 5,6-fused rings and the very strained cyclobutanol moiety with exact relative stereochemistries, respectively.

**Scheme 23:**
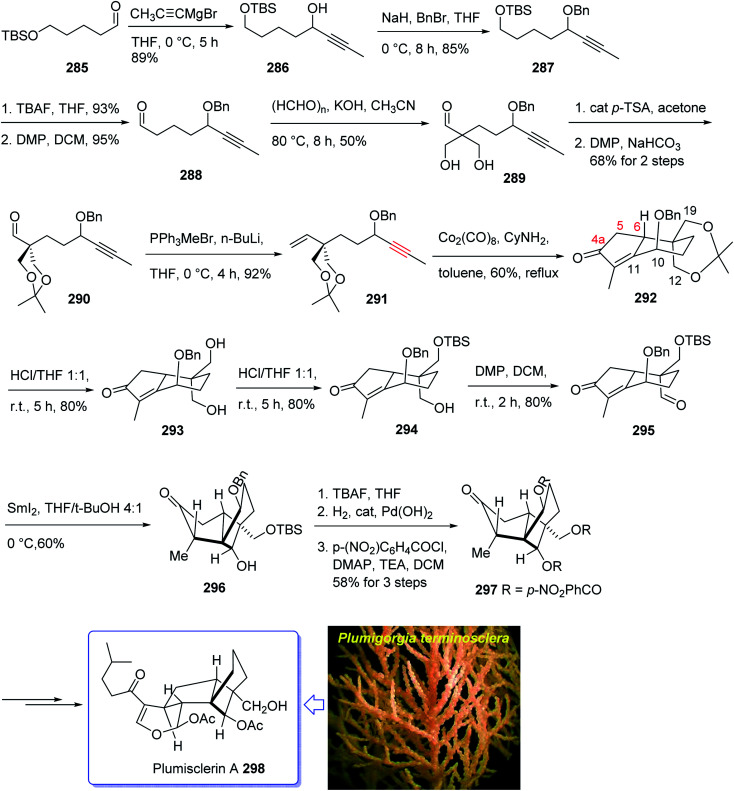
Total synthesis of plumisclerin A 298.

This attempt started with alcohol 286 which in turn is provided from the addition of propynyl magnesium to the known aldehyde 285.^202^ Alcohol 286 is converted after several steps to enyne 291 as a suitable precursor of PKR *via* various functional group transformations as well as protecting–deprotecting processes. Having enyne 291 available in hands, it was investigated in a PKR^5,7^ explaining various combinations to find an optimal reaction conditions. Eventually, enyne 291 was converted to the desired compound 292 in modest yield when PKR was catalyzed by Co_2_(CO)_8_ in the presence of cyclohexylamine (CyNH_2_). Worthy to mention that, the C10-OBn of enyne 291 in PKR, played a vital role to control the newly generated chiral center at C6 position, when the requisite cyclopentenone ring was constructed. Then, compound 292 was converted to compound 295 in three steps in which the latter upon treatment with SmI_2_ in THF and *t*-BuOH (4 : 1, v/v) as mixed solvents at 0 °C afforded the anticipated bridged-compound 296 in good yield. After three steps, the latter was unambiguously transformed into the respective tri-*p*-nitro-benzoate 297 (confirmed by single crystal X-ray analysis). The latter was then transformed to the desired plumisclerin A 298 after several steps. In conclusion, an efficient asymmetric synthesis of the tricyclo [4,3,1,0^1,5^] decane core of cytotoxic marine diterpenoid plumisclerin A 298 was successfully achieved in several steps from the easily accessible ω-hydroxypentanal 285 (Scheme 23).^201^

Marrulibacetal 315, a diterpenoid, was initially isolated from the aerial parts of *Marrubium globosum* ssp. *libanoticum* by Borrelli *et al.* in 2009.^203^ The same group reported the structural elucidation of marrulibacetal 315 in the same year.^203,204^*Marrubium globosum* ssp. *libanoticum* have been used for a long time as medicinal plant which are used as hypoglycemic, febrifuge, antispasmodic, and anti-inflammatory drugs in Northern Lebanon.^203^

**Scheme 24:**
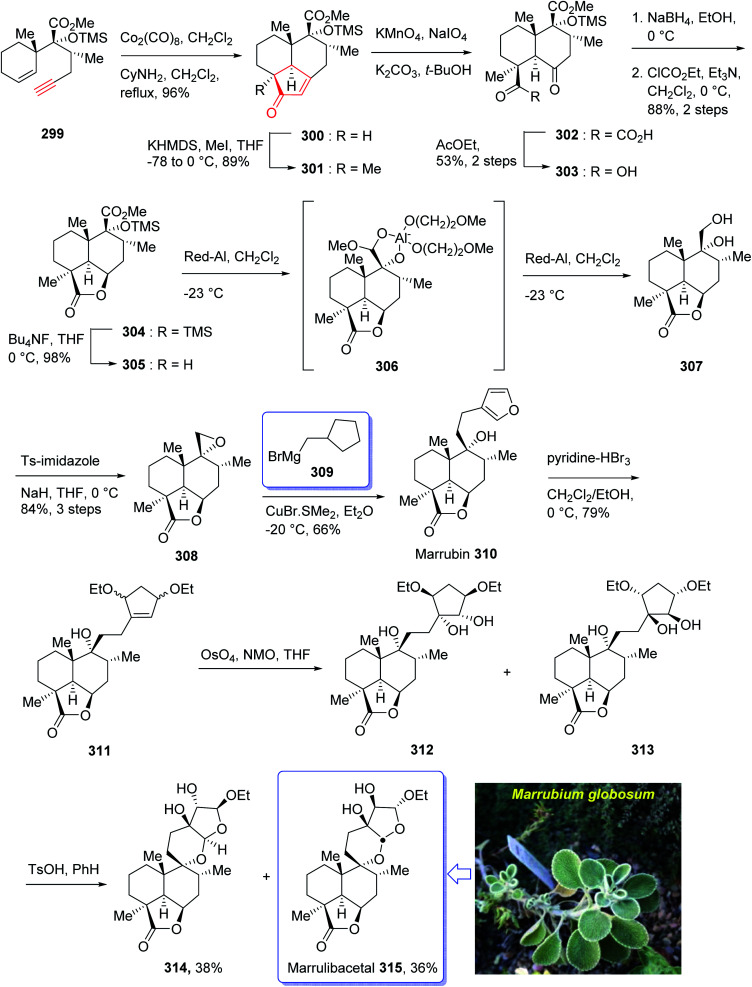
Total synthesis of marrulibacetal 315.

In 2016, Nakamura *et al.*^205^ achieved and reported a stereoselective total synthesis of (+)-marrubiin commencing from a chiral scaffold *via* the CyNH_2_-catalyzed Pauson–Khand reaction followed by oxidative cleavage of the resultant cyclopentenone ring. This strategy started from enyne 299 (ref. 206) which was subjected to PKR using Co_2_(CO)_8_ in CH_2_Cl_2_ at ambient temperature with subsequent addition of cyclohexylamine, followed by dilution with dichloromethane, and refluxing the mixture to afford tricyclic enone 300. The latter was transformed to a 1 : 1 diastereomeric mixture of *cis*-diols 312 and 313 after several steps, including various functional group transformations. Ultimately, internal transacetalization of *cis*-diols 312 and 313 in the presence of TsOH in benzene gave the desired natural product, (−)-marrulibacetal 315, along with its isomer (Scheme 24).^205,207^

**Scheme 25:**
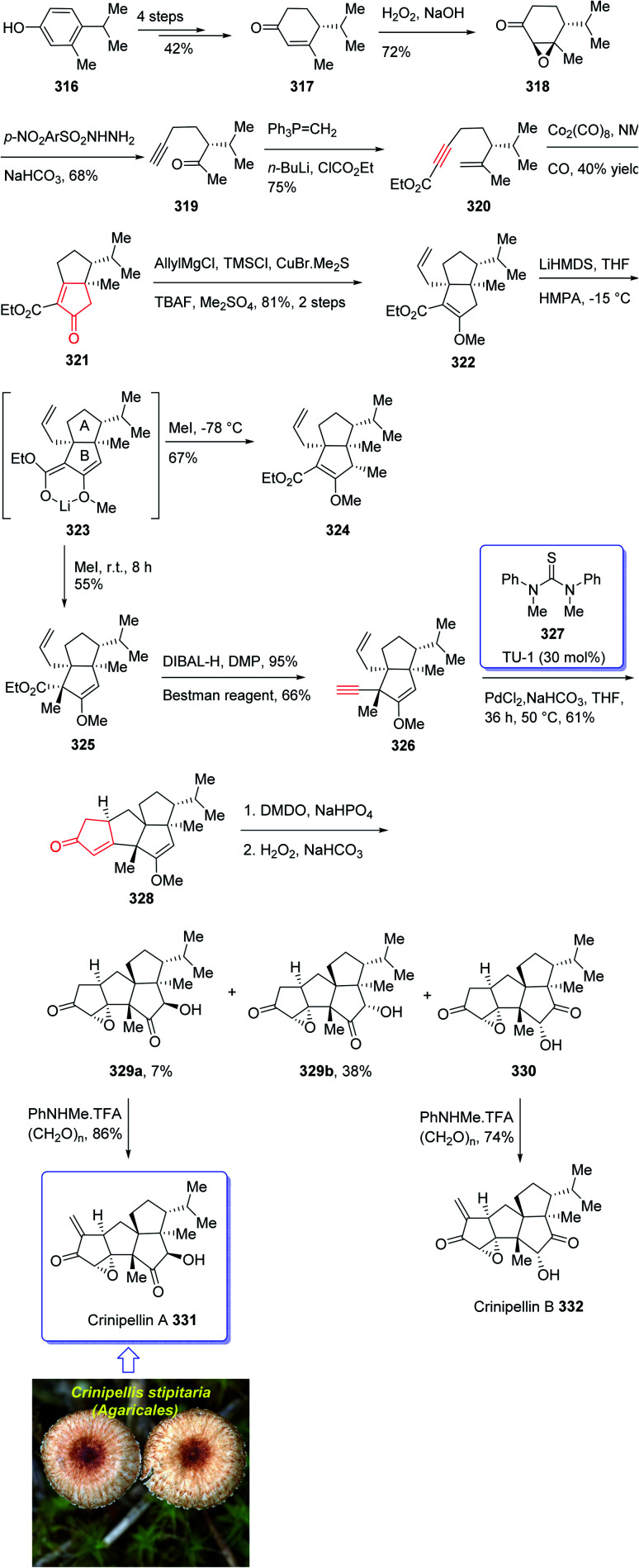
Total synthesis of crinipellin A 331, crinipellin B 332.

Crinipellin A 331 is classified as diterpenoid.^208^ It was isolated and reported in 1979 by Steglich *et al.* from the fungus *Crinipellis stipitaria* (Agaricales).^208,209^ It has an interesting chemical structure bearing α-methylene ketone moiety and an exceptional tetraquinane core, containing eight chiral centers, in which three of them are adjoining all-carbon quaternary carbons. From biological points of view, 331 and 332 were found to exhibit antibiotic potencies.^208,209^ The total synthesis of racemic 332 was accomplished in twenty-two steps *via* Barbier annulation by Piers and co-workers and reported in 1993.^210^ In 2014, Lee and co-workers achieved and reported the first asymmetric total synthesis of 331 through a tandem [3 + 2] cycloaddition reaction for the construction of its tetraquinane scaffold containing three successive quaternary chiral centers.^136,211,212^ In 2018, Yang and co-workers^213^ accomplished and reported the asymmetric total syntheses of (−)-crinipellin A 331 and (−)-crinipellin B 332 in eighteen steps from the commercialized phenol 316, respectively. These total syntheses featured a developed thiourea/Pd-catalyzed intramolecular Pauson–Khand reaction for the asymmetric construction of the tetraquinane scaffold present in crinipellins. As a matter of fact, the vital step was PKR which provided compound 321 containing two *cis*-configured vicinal chiral centers.^214^ The required enyne ester 320, as a required precursor of PKR, which was provided *via* the Trost methodology^215^ from already known compound (R)-4-isopropyl-3-methylcyclohex-2-en-1-one 317. The latter in turn was synthesized in four steps from market purchasable 4-isopropyl-3-methylphenol 316.^216^ Having compound 318 in hand, it was subjected to an asymmetric Weitz–Scheffer-type epoxidation,^217^ to give ketone 319 which was condensed with *p*-NO_2_ArSO_2_NHNH_2_ followed by treatment with NaHCO_3_ to undergo Eschenmoser fragmentation^218^ to give the acetylene ketone 319 in 68% yield. The latter first underneath a Wittig reaction, and the resultant enyne, upon sequential direct treatment with BuLi and ethyl chloroformate afforded enyne 320 as a suitable precursor for PKR. This one-pot transformation is essential to ensure a high yield because the intermediate enyne is volatile. After considerable experimentation, it was found by a crucial step, enyne 320 was treated with a stoichiometric quantity of Co_2_(CO)_8_ at ambient temperature for a while and then the resultant enyne/Co complex was gradually heated to 76 °C in the presence of 4-methylmorpholine *N*-oxide (NMO) for relatively long time (36 h), to obtain 322 in modest yield but excellent ee (98% ee) after crystallizations. This obtained ketoester 322 was reacted with an organocopper reagent (provided from treatment of allylmagnesium chloride and CuBr·Me_2_S^219^ at −78 °C) proceeded *via* highly diastereoselective conjugate addition reaction, to give the anticipated enolate which was reacted with Me_2_SO_4_ in the presence of Cs_2_CO_3_ and tetrabutylammonium fluoride (TBAF)^220^ to afford methyl vinyl ether 322 as a sole isomer in satisfactory yield. On the other hand, compound 323 was converted to another key intermediate 325, in two steps involving regio- and stereoselective assemblage of quaternary chiral center. This vital intermediate ester 325 was reduced with DIBAL-H, with subsequent oxidation of the resultant using the Dess–Martin reagent followed by reaction with the Bestmann reagent^221^ to afford enyne 326 as a suitable PKR precursor in good yield. Enyne 326 was then subjected to Pd-catalyzed PKR for the successful construction of tetraquinane 328. It is worthwhile mentioning that use of TU-1 ligand 327 (ref. 222) improved the diastereoselectivity of this PKR affectedly. Treatment of 328 with dimethyldioxirane (DMDO) in a Na_2_HPO_4_ solution^223^ and H_2_O_2_/NaHCO_3_ in sequence afforded the epoxides 329a and 329b after several steps in 7% and 38% yields, respectively. Then, compound 329a was subjected to modified Eschenmoser methylenation upon treatment with *N*-methylanilinium trifluoroacetate and paraformaldehyde in THF at 70 °C to afford the desired natural product crinipellin A 331 in 86% yield.^209^ Having 329b in hands, it was converted to the thermodynamically stable compound 330*via* isomerization^224^ of its α-hydroxy ketone motif. This isomerization of 329b to 330 was successfully accomplished by treatment of 329b with various acidic and basic reagents. Compound 330 was then treated with *N*-methylanilinium trifluoroacetic acid (TFA) and paraformaldehyde in THF at 70 °C to afford the other natural product, crinipellin B 332 in 74% yield (Scheme 25).^213^

### 2.4. Sesquiterpenes

**Scheme 26:**
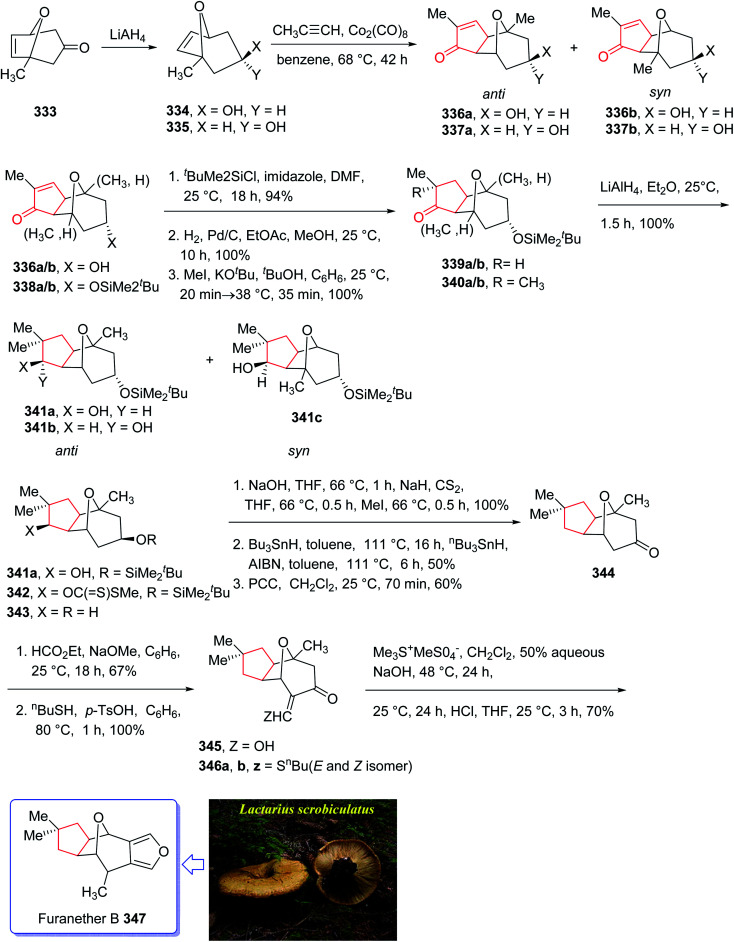
Total synthesis of furanether B 347.

Furanether B 347, a member of the lactarane class of sesquiterpenes, was initially isolated by Vita-Finsi and co-workers in 1980.^225^ The total synthesis of furanether B 347 was achieved and reported by Schore in co-workers in 1989 (ref. 226) using PKR as a key step. This strategy started with ketone l-methyl-8-oxabicyclo [3.2.l] oct-6-en-3-one 333, provided *via* a reported method by Noyori *et al.* from 2-methylfuran and tetra-bromoacetone.^227^ Ketone 333, upon reduction by lithium aluminum hydride as reducing agent gave *exo* : *endo* isomeric alcohols 334 and 335 in 2 : 1 ratio and in 94% yield. Stereoisomeric alcohols 334 and 335 were reacted with propyne under PKR conditions (Co_2_(CO)_8_, benzene, heat) to afford a mixture of four isomeric tricyclic ketones (336a, 336b and 337a, 337b). Pauson–Khand cycloaddition by reduction of 333 with lithium aluminum hydride. Pauson–Khand cycloaddition of propyne and the mixture of stereoisomeric alcohols 334 and 335 gave 75% yield of a mixture of four isomeric tricyclic ketones (336a, 336b and 337a, 337b). Among them, 336a was converted to ketone 344 after several steps. The latter was then formylated regiospecifically to afford compound 333 in good chemical yield.^228^ In solution, 345 contains 75–80% intramolecular hydrogen-bonded (*Z*)-β-hydroxyenone, the remainder being the *E*-isomer and traces of ketoaldehyde. Compound 345 was subjected to Ireland's procedure for the synthesis of the thiomethylene derivative to give 346a and 346b in almost quantitative yield.^229^ This mixture with treated with trimethylsulfonium methylsulfate in a two-phase system to give an epoxide that upon rearrangement on standing at ambient temperature for 24 h (ref. 230) followed by aromatization in the presence of HCl in THF gave the desired natural product, furanether B 347 in moderate yield.^231^ Spectral data obtained for synthetic 334 were in agreement with those obtained from the isolated natural product (Scheme 26).^226^

**Scheme 27:**
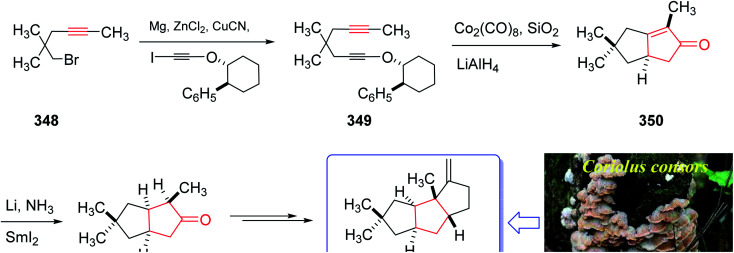
Total synthesis of hirsutene 352.

The mold metabolite, hirsutene 352, is parent member of an important class of linear triquinane sesquiterpene. It was initially isolated from the hydrocarbon extracts of fermented mycelium of *Coriolus consors* by Nozoe and co-workers in 1976.^232^ Hirsutene 352 did not show any significant biological potency whereas its derivative, diketocoriolin B, exhibited a prominent cytotoxic,^233^ antibiotic and antitumor potencies.^234^ Therefore, its total synthesis attracted much attention of synthetic organic chemists.^234^ Hirsutene 352 was fully characterized by analysis of combined data, obtained from various spectroscopic techniques, commonly used for the structural elucidation of organic compounds.^232^ In 1990, Pericas and co-workers^235^ achieved and reported an efficient total synthesis of hirsutene 352 using intramolecular Pauson–Khand reaction.^232^ Homochiral diyne 352 was easily synthesized from coupling of 348*via* a Cu-mediate coupling reaction^236^ involving the zinc reagent. In this strategy, enyne 349 as an appropriate PKR precursor was converted initially to the respective *E*-enol ether by treatment of the latter with LiAIH_4_ in THF and the subjection of the resultant to PKR conditions (Co_2_(CO)_8_, SiO_2_, purification, 85%) as key bi-cyclization under mild reaction conditions^5,237^ to afforded enone 350 diastereoselectively. The latter upon either Birch reduction or less effectively, catalytic hydrogenation^238^ gave 351 in racemic form. This bicyclic ketone 351, was transformed to the desired natural product hirsutene 352 in several steps following the previously reported procedure (Scheme 27).^235^

(+)-Taylorione 365 is the pure enantiomer of the principal sesquiterpene isolated from extract of the common leafy liverwort *Mylia taylorii*^239^ found on the northern hemisphere. The structure of (+)-taylorione 365 was elucidated and its absolute configuration was determined by combination of its spectral data analysis and degradation investigation.^240^

**Scheme 28:**
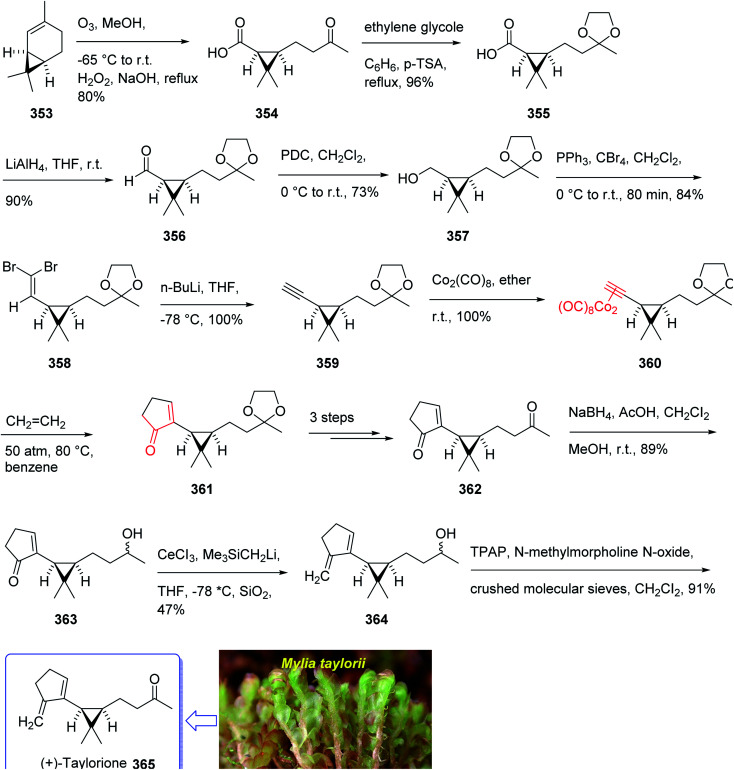
Total synthesis of (+)-taylorione 365.

The total synthesis (+)-taylorione 365 was accomplished and reported by Kerr *et al.* in 1996 (ref. 241) using the Pauson–Khand annulation reaction as the key step. It began from already prepared (+)-2-carene 353 (ref. 241 and 242) which upon ozonolysis and subsequent oxidation with basified hydrogen peroxide, gave the keto acid 354 in 80% yield. The latter was converted to the ketal 351 upon the reaction with ethylene glycol in the presence of *p*-TSA in refluxing benzene. Since compound 355 found to be unstable to storage, it was instantly reduced to the corresponding alcohol 356 by treatment with LiAIH_4_ in THF followed by oxidation with PDC to the corresponding aldehyde 357 in 73% yield. Having aldehyde 353 in hand, the synthesis of the essential alkyne complex 360 was contemplated. The latter was treated with CBr_4_ in CH_2_Cl_2_ to obtain the desired dibromo olefin 358 in 84% yield in pure form after column chromatography. The dihalo alkene 358 was transformed to the pure terminal alkyne 359 rapidly upon treatment with *n*-BuLi followed by aqueous work up. Then, alkyne 359 was reacted with octacarbonyldicobalt at ambient temperature to afford the dicobalt complex 360 in almost quantitative yield. The alkyne complex 360 was then underwent PK annulation reaction (C_2_H_4_, 50 atmospheres, 80 °C, benzene) to afford the desired cyclopentenone 361 in 38%. Pleasantly, upon treatment of cyclopentenone 361 with PPh_3_ and CCl_4_ in CH_2_CI_2_ at 0 °C to ambient temperature, the required diketone 362 was provided in an excellent yield. Having 362 available in hands, the remaining steps in the synthesis proceeded with no complication. The latter was subjected to selective carbonyl reduction under Ward conditions^243^ (NaBH_4_ in CH_2_C1_2_/AcOH/MeOH) to provide the hydroxy ketones 363. After conversion of the latter to the diene 364 in moderate yield, to obtain optimum oxidation, it was reacted with Griffith–Ley tetrapropylammonium perruthenate (TPAP) agent to obtain the desired natural product 365 in 91% yield. In conclusion, the total synthesis of enantiopure (+)-taylorione 365 was accomplished starting from readily accessible chiral pool reagent (+)-2-carene 353, in a brief manner (in twelve steps) in a good overall yield of 12% (Scheme 28).^241^

(+)-Pentalenene 375 (1*R*,3a*S*,5a*S*,8a*R*)-1,2,3,3a,5a,6,7,8-octahydro-1,4,7,7-tetramethyl cyclopenta[*c*]pentalene, was initially isolated by Seto and co-workers in 1980 (ref. 244) from the extract of *Streptomyces griseochromogenes*. Its structure elucidation revealed it has angularly fused triquinanes.^245,246^ This tricyclic sesquiterpene 375 is involved in the biosynthesis of neopentalenolactone antibiotic.

Its total synthesis has been benchmark of various strategies involving the regio- and stereo-selective assembly of cyclopentanoid systems.^246,276^

**Scheme 29:**
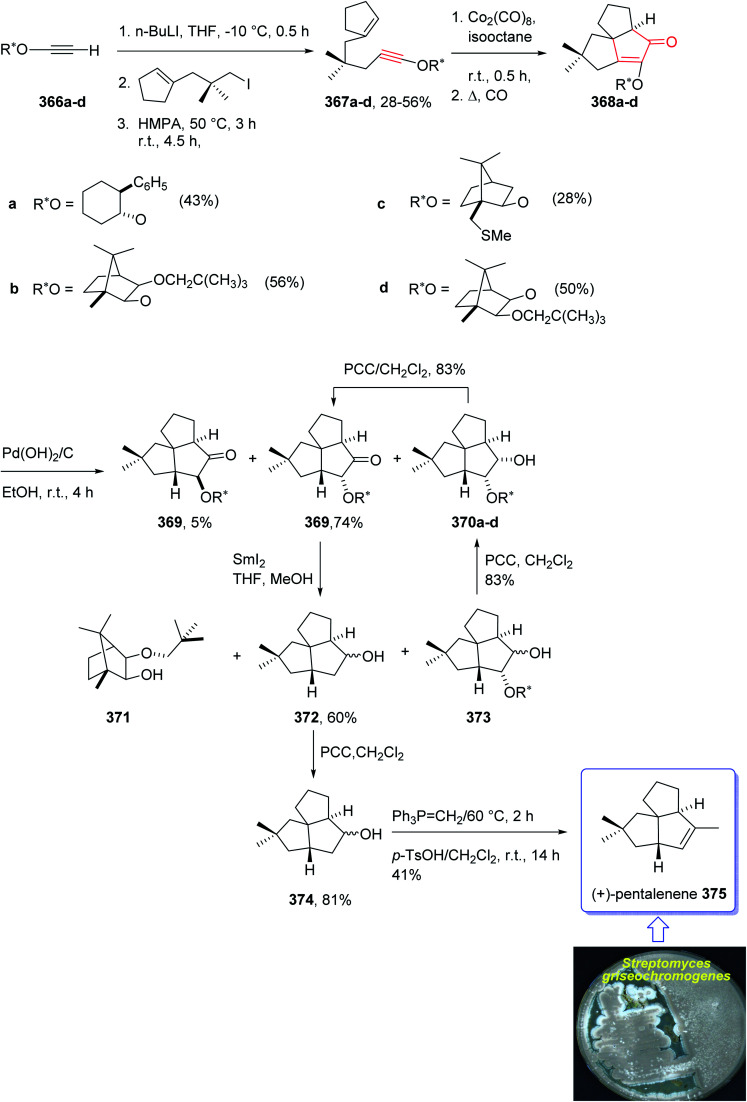
Total synthesis of (+)-pentalenene 375.

In 1997, Moyano and co-workers achieved and reported an effective total synthesis of 375 using Pauson–Khand reaction as key step.^247^ Accordingly, the total synthesis started from lithium acetylide derived from (±)-(*trans*-2-phenylcyclohexyloxy)ethyne 366a, created in dry tetrahydrofuran as solvent to furnish in enyne 367a in moderate yield. The latter was subjected to a diastereoselective intramolecular Pauson–Khand reaction, following the procedure, already reported by Schore *et al.* who have synthesized the triquinane system of racemic 375 (ref. 245) in this way, the enyne 367 underwent a diastereoselective cyclization to provide the key intermediate 368. Noticeably, it became evident that for such diastereoselective Pauson–Khand reaction, the best choice of chiral auxiliary is 3-(neopentyloxy)isoborneol. After several steps involving various functional group transformations, compound 368 converted to ketone 374 as illustrated in (Scheme 29). The configuration of 374 was determined from absolute configuration of 368c, 369, 370, and 372 thus, had been established, unambiguously.

In the final step of this total synthesis, ketone 374 was transformed into the desired natural product, (+)-pentalenene 375, *via* Wittig olefination and acid-promoted isomerization of the exocyclic double bond (Scheme 29).^248^

Sesquiterpene, illudin S, exhibited high antitumor activity.^249^ Later, illudin analogues were synthesized showing highly improved effectiveness in comparison with the parent compounds.^250^ One of such analogues is hydroxymethylacylfulvene 381 (HMAF, also called MGI 114). Since it was found to be active against breast, lung, and colon tumors, it has attracted much attention of synthetic organic chemists while showing intensely abridged toxicity. In addition, HMAF^251^ as also found to be potent towards the MDR phenotype.^252^

The hydroxymethylacylfulvene 385 can be provided semi-synthetically from the naturally occurring sesquiterpene illudin S. Illudin S is generated in cultures of *Omphalotus illudens* (Jack o'-lantern mushroom). Upon treatment of illudin S with formaldehyde in 1 N H_2_SO_4_ solution, HMAF can be obtained through a reverse Prins reaction to give the intermediate acylfulvene 384 which next can be subjected to an ene reaction with formaldehyde.^253^ The first total synthesis of HMAF was achieved and reported by McMorris *et al.* in 1997 (ref. 254) comprising a Padwa kind carbonyl ylide 1,3-dipolar cycloaddition^255^ to achieve the illudin scaffold.

**Scheme 30:**
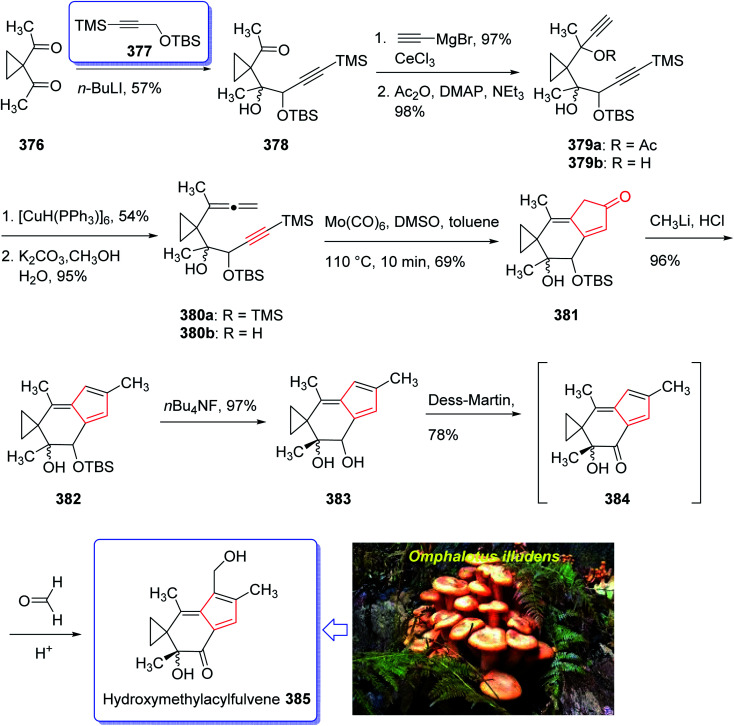
Total synthesis of hydroxymethylacylfulvene (HMAF) 385.

A brief synthetic approach involving eleven steps of HMAF was accomplished and reported by Brummond and co-workers in 1999,^256^ including an intramolecular [2 + 2 + 1] Pauson–Khand reaction. In this strategy, the easily accessible 1,1-diacetylcyclopropane 376,^256^ reacted with the lithio derivative of the *tert*-butyl-dimethylsilyl ether of 3-trimethylsilylpropyn-1-ol 377 to give the expected ketone 378 as a 1.3 : 1 mixture of diastereomers in good yield. After several steps, the latter was transformed into alkynyl allene 380b which was subjected to fast cycloaddition under the conventional PKR conditions [Mo(CO)_6_, DMSO, toluene, 110 °C]^257,258^ to afford the 4-alkylidene cyclopentenone 381 as single product in good yield. The latter was transformed into the secondary alcohol 383 after several steps which was oxidized using Dess–Martin oxidative reagent to the corresponding ketone, acylfulvene 384, in good yield. The latter was then reacted with formaldehyde in the presence of H_2_SO_4_ in acetone/water to give HMAF 385 in good yield (Scheme 30).^256^

Recently, asteriscanolide 404 which is a cyclooctane sesquiterpene lactone has attracted much attention of organic synthetic chemists. It was initially isolated in 1985 by the Feliciano research group from the hexane extract of *Asteriscus aquaticus*.^259^ Several successful attempts have been reported. Wender and co-workers,^260^ the Paquette group^261^ and very recently the Snapper research group^262^ have reported the total synthesis of asteriscanolide 404.

In 2001, Krafft *et al.* reported an intermolecular Pauson–Khand cycloaddition and a ring-closing metathesis as vital steps.^263^ This strategy incorporates the cyclooctane chiral center prior to ring construction. Remarkably, the ring-closing metathesis creates a new eight-membered ring with an “in–out” intrabridgehead relationship which come across the principles as mentioned above.

**Scheme 31:**
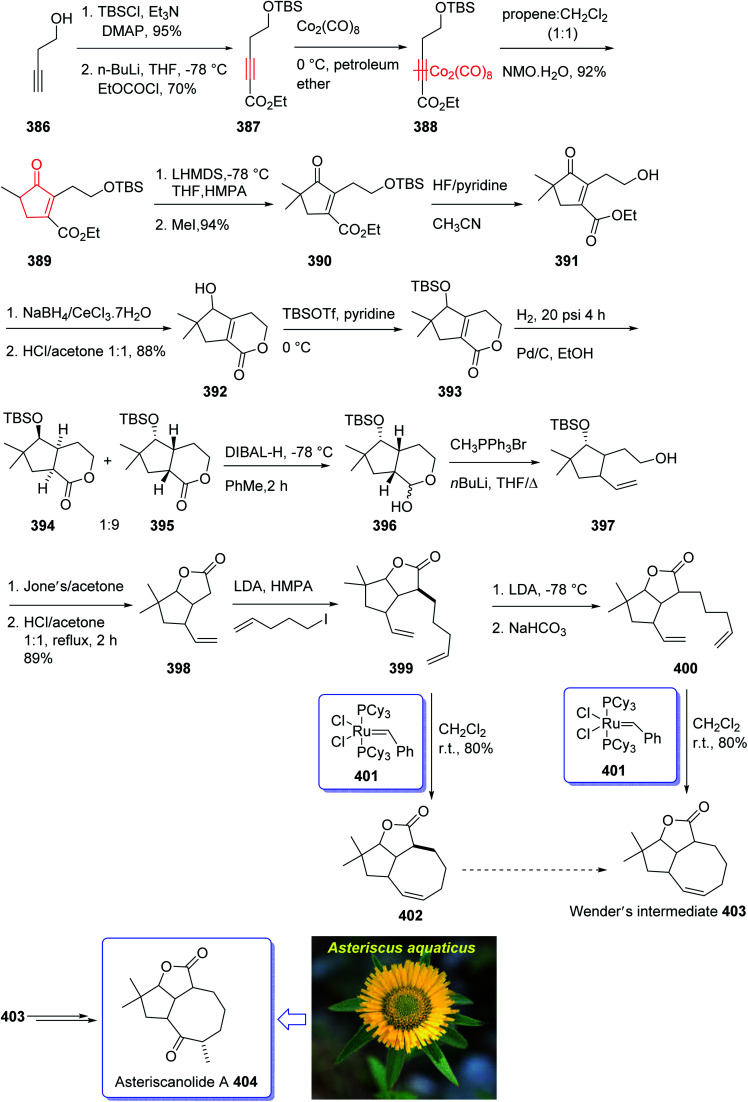
Total synthesis of asteriscanolide 404.

The total synthesis started from the protected 3-butyn-1-ol 386 as the corresponding *tert*-butyldimethylsilyl (TBS) ether which was converted into the corresponding alkynoate 387*via* treatment with *n*-BuLi in THF at −78 °C to create the lithio alkyne and then added to ethyl chloroformate in THF at −78 °C. Treatment of 387 with dicobalt octacarbonyl in petroleum ether gave the desired precursor for PKR, hexacarbonyl-dicobalt complexed alkyne 388. The latter was then reacted with propene in methylene chloride followed by incremental addition of *N*-methyl-morpholine-*N*-oxide monohydrate under PKR conditions. This afforded the highly functionalized cyclopentenone 389 which comprises different functional groups suitably placed for further employing of the side chains. After several steps, the latter was converted into trisubstituted cyclooctene 403. The latter was a key intermediate for the successful total synthesis of desired natural product, asteriscanolide A 404, after several steps (Scheme 31).^263^

**Scheme 32:**
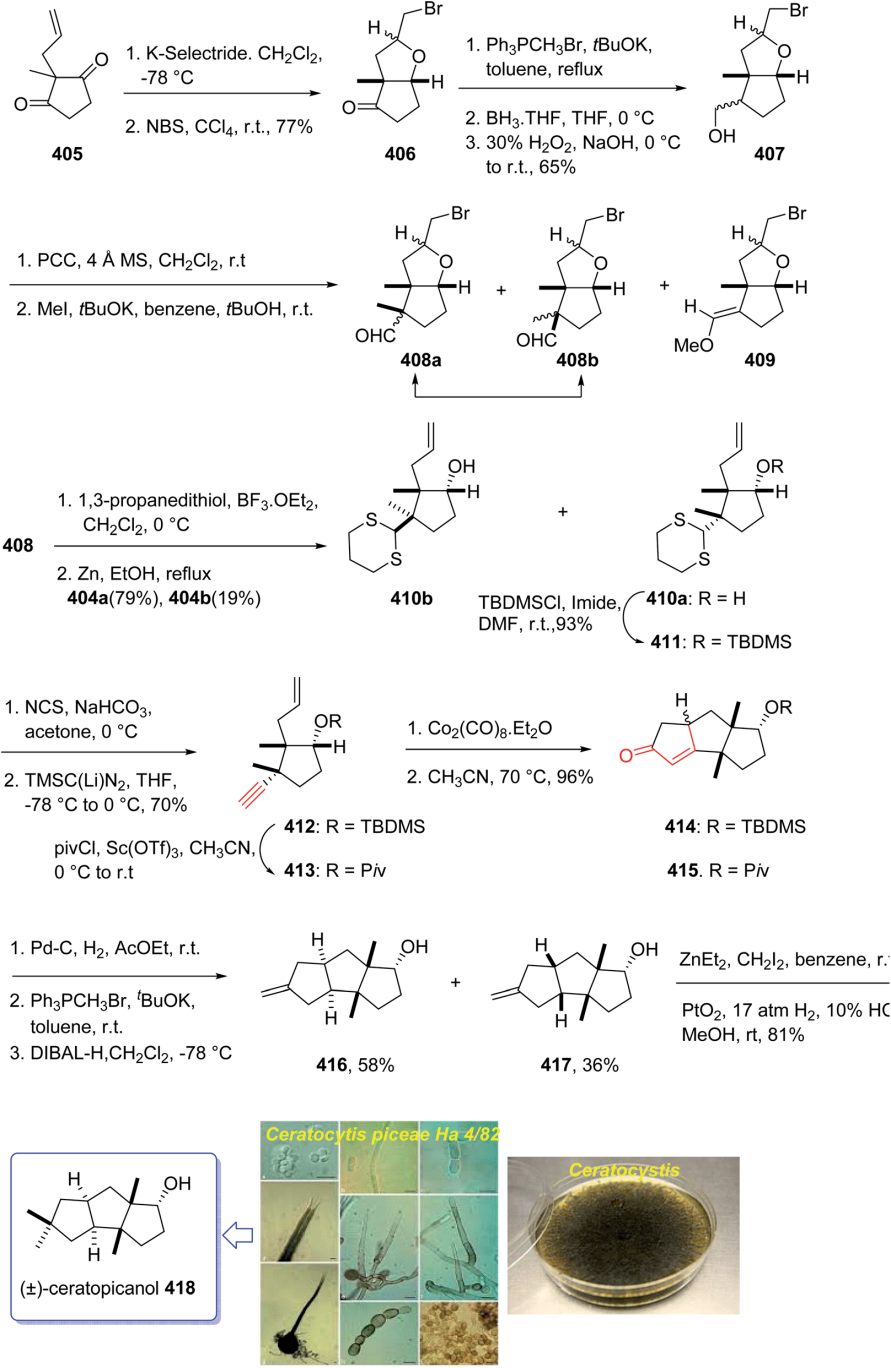
Total synthesis of (+)-ceratopicanol 418.

Optically active (+)-ceratopicanol 418, as a novel triquinane sesquiterpene was initially isolated from the extract of fungus *Ceratocystis piceae* Ha 4/82 (ref. 264) by Hanssen and co-workers in 1998.^264^ Its structure was elucidated and its relative stereochemistry was determined as (1R*,2S*,6S*,8S*,9R*)-1,4,4,8-tetramethyltricyclo[6.3.0.0^2,6^]-undecan-9-ol.^264^ The absolute configuration of (+)-ceratopicanol 418 was determined unambiguously when the total synthesis of its unnatural (−)-stereoisomer was completed and its X-ray analysis was compared to the (+)-natural product 418.^265^ Ceratopicanol 418 has a fascinating and exceptional structural characteristic containing five stereogenic centers among which are two adjoining bridgehead quaternary carbon centers. Due to these exceptional features, ceratopicanol 419 was selected as a target to be synthesized by several research groups.^265,266^ In 2002, Mukai *et al.*^267^ studied the stereoselective reduction of the 1,3-dicarbonyl 405 resulting in the formation of a compound containing hydroxyl and allyl moieties with *cis*-relationship. They found out when compound 405 is treated with *K*-selectride, the highest stereoselectivity was obtained. To remove the undesired *trans*-hydroxy compound, a mixture obtained from reduction step was subsequently treated with *N*-bromosuccinimide (NBS) in CCl_4_ to provide a mixture containing two stereoisomers of the 2-oxabicyclo[3.3.0]octa-6-one derivative 406, because of the presence of C_3_ stereogenic center, in satisfactory overall yield. After the next steps including different functional group transformations such as treatment of 408 with 1,3-propanedithiol in the presence of BF_3_·OEt_2_ which provided separable 410a (75%) and 410b (19%), conversion of secondary hydroxyl group of 410a to 411 in 93% yield, followed by treatment with lithiotrimethylsilyldiazomethane, the alkyne derivative 412 was obtained as key intermediate. In accordance with the classical Pauson–Khand reaction procedure, compound 412 was treated with [Co_2_(CO)_8_] in Et_2_O to give the corresponding alkyne–cobalt complex. This complex upon heating at 70 °C in CH_3_CN^268^ gave compound 414 in high yield. Then, to a pivaloyl group was introduced on the secondary hydroxyl group of 412 to produce 413. After treatment with Co_2_(CO)_8_, the latter was transformed to the corresponding cobalt-complex 415. Elimination of the pivaloyl group resulted in the isolation of the desired compound 416 together with 415. Finally, compound 416 in pure form, treated with diethyl zinc and diiodomethane in benzene under the Simmons–Smith reaction to obtain the cyclopropane derivative which upon hydrogenation in the presence of PtO_2_ under pressure gave the desired natural product (±)-ceratopicanol 418 in 81% yield (Scheme 32).^267^

The tricyclic sesquiterpenes α-cedrene 428a and β-cedrene 428b were initially isolated by Barrero *et al.*,^269^ in 1996 from *Juniperus cedrus* and *Juniperus thurifera*.^269–271^ With these natural products, α-cedrene 428a and β-cedrene 428b, a range of accurately relative oxygenated terpenoid analogues were also isolated from the same source. Due to their fascinating [5.3.1.0^1,5^] tricyclic structure, the cedrene family and their relative naturally occurring compounds have attracted much attention of synthetic organic chemists over the years since initial characterization of α-cedrene 428a and β-cedrene 428b in 1953 by Stork research group.^272^

In 2006, Kerr and co-workers^273^ developed a pathway involving an intramolecular Pauson–Khand reaction in which the cedrene carbon scaffold was effectively installed from a simple monocyclic precursor directly.

A small number of further synthetic manipulations provided a concise formal total synthesis of α- and β-cedrene. The cyclisation precursor was readily prepared with a stereoselective ketone alkenylation selectively providing the olefin required for efficient access to the natural target. Using a simple monocyclic precursor led to the direct and highly effective formation of the interesting tricyclic [5.3.1.0^1,5^] carbon scaffold of α-cedrene 428a (and β-cedrene 428b). It is noteworthy that in the crucial key Pauson–Khand annulation step, the olefinic precursors reacted with retention of configuration to afford the expected cyclopentenone epimer for synthesis of the desired natural products in excess. For further magnifying the effectiveness of this total synthetic design, the remaining and undesired cyclopentenone was also transformed into the vital isomer by a simple base-prompted epimerization course. In sequence, the desired cyclopentenone intermediate was further expanded to cedrone 427, therefore, establishing a formal total synthesis of α- and β-cedrene. Ultimately, it is worthwhile to notice that compound 425a underneath a reaction sequence comparable to those conducted on 425b resulting in the synthesis of *epi*-cedrone.

**Scheme 33:**
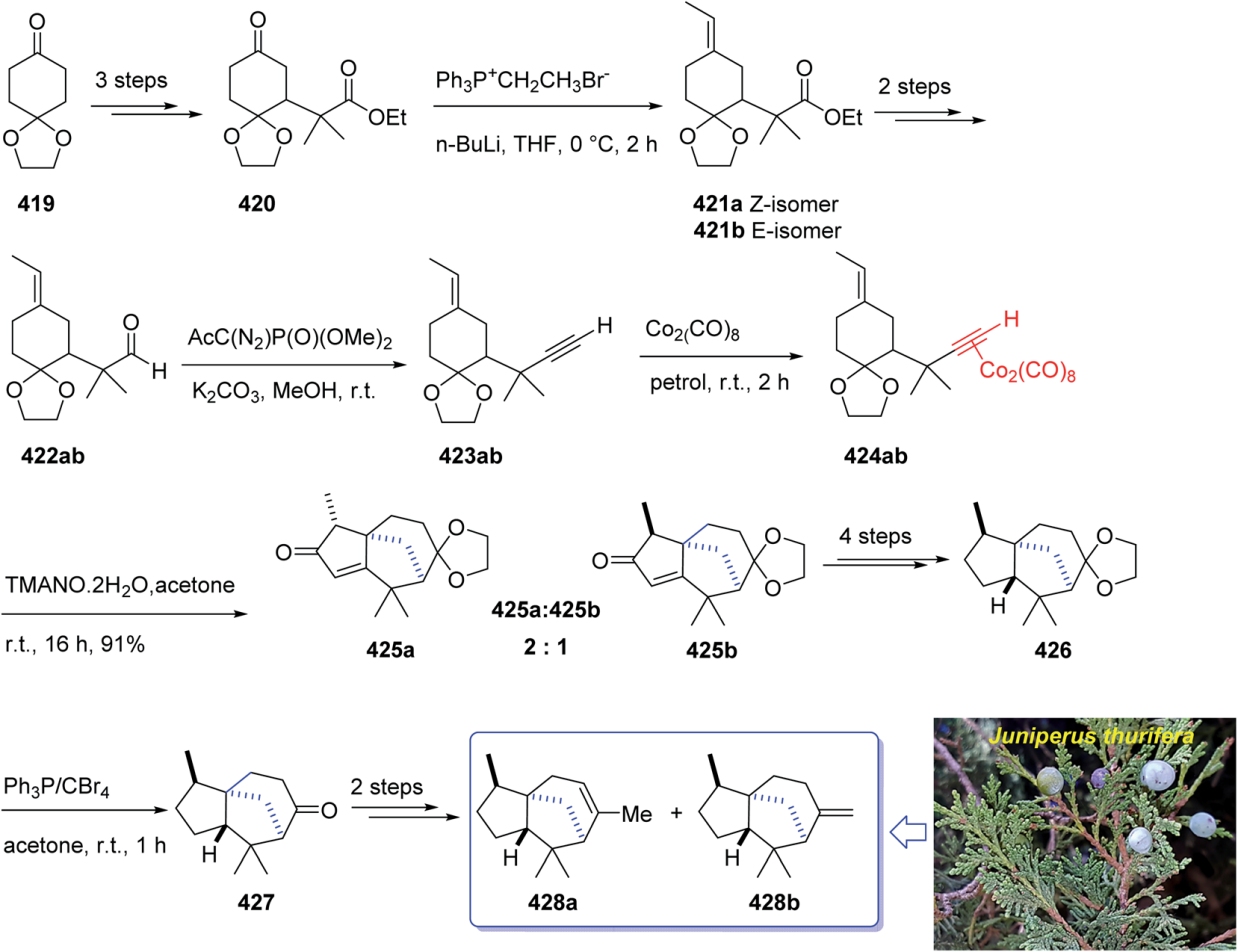
Total synthesis of α-cedrene 428a and β-cedrene 428b.

**Scheme 34:**
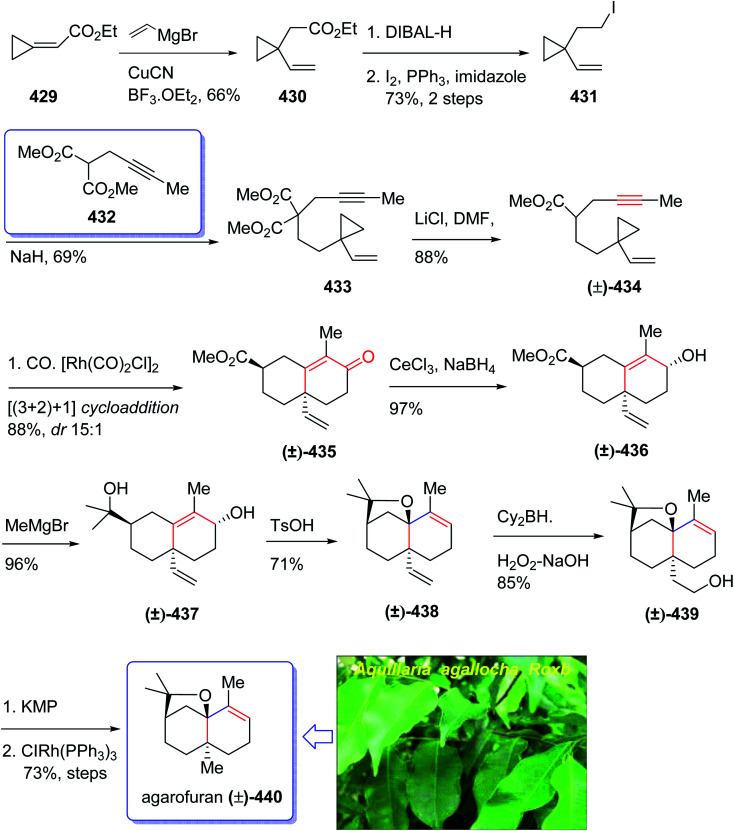
Total synthesis of (±)-α-agarofuran 440.

As a matter of fact, this synthetic pathway is commenced with the introduction of α,β-unsaturation into the market purchasable cyclohexanedione monoethylene acetal 419. After three steps, compound 419 was converted into 420*via* Saegusa oxidation. The latter was then subjected to standard Wittig reaction at 0 °C employing the ethyltriphenylphosphonium bromide salt and *n*-BuLi to give olefins 425a/425b in 92% yield as a 2 : 1 mixture of geometric isomers. The compound 421a/421b was transformed into aldehydes 422ab in 97% yield by oxidation after two steps.^274^ For the transformation of aldehydes 422ab into alkynes,^221,275^ the Ohira–Bestmann technique (with the reagent dimethyl acetyldiazomethyl-phosphonate) was applied. In this case, this mild strategy gave alkynes 423a/423b in 81% yield. In sequence, these were habitually complexed with octacarbonyldicobalt to provide the stable cyclisation precursors 424a/424b in a virtually quantitative yield. At this vital step and with the essential complexes in hand, the Pauson–Khand annulation for the installation of the required tricyclic carbon α-cedrene scaffold was examined. Delightfully, intramolecular Pauson–Khand cyclisation of 424a/424b proceeded smoothly to afford the enones 425a/425b in high yield as a mixture of stereoisomers in the ratio of 2 : 1. This indicated that relative stereochemistry present in the initial olefins 421a/421b had been carried *via* the cyclisation of precursors 424a/424b. Using an efficient and selective approach to 425b, an essential deoxygenated product 426 was provided after four steps. Upon treatment of 426 with Ph_3_P/CBr_4_, desired cedrone 428 was obtained in 99% yield.^276^ Then, the latter in two steps was converted to desired natural product α- and β-cedrene, 428a and 428b (Scheme 33).^273^

(±)-α-Agarofuran 440 is a racemic furanoid sesquiterpene natural product. In 1962, Bhattacharyya *et al.*,^277^ isolated α- and β-agarofuran from extract of agarwood C (*Aquilaria agallocha Roxb*).^278^ Its structure was elucidated by chemical degradation and spectroscopic data analysis, chemical examination of fungus infected agarwood C (*Aquilaria agallocha Roxb*).^279,280^ An approach to the total synthesis of α-agarofuran including Pauson–Khand reaction as a key step was presented by Yu *et al.* in 2010.^281^ This strategy started from cyclopropylidene ester 429,^282^ which in two steps using conventional organic reactions, was converted to vinyl cyclopropane iodide 431. Next, the latter was coupled with propargyl diester 446 to afford the *gem*-diester-tethered 1-yne-VCP 433. Then, the latter was subjected to Krapcho decarboxylation^283^ to provide monoester-substituted 1-yne-VCP 434. This compound can act as a suitable precursor for homo-Pauson–Khand cycloaddition reaction under already secured optimal reaction conditions to give bicyclic cyclohexenone 435 in 86% isolated yield with a good diastereoselectivity. Compound 438 having the tetrahydrofuran framework of agaroguran,^284^ was synthesized from 435 after several steps. Ultimately, the vinyl unit was converted respectively to its corresponding alcohol and then angular methyl group by a sequential hydroboration–oxidation–decarbonylation to afford the desired natural product as a racemat (±)-α-agarofuran 440 (Scheme 34).^280,281^

Kitanaka^285^ and co-workers in 2005 for the first time isolated a new guaiane-type sesquiterpene, (+)-indicanone 454, from the extract root of *Wikstroemia indica* (Thymelaeceae), collected from the southeast China. Notably, in this isolation, two already known and reported biflavonoids (*i.e.* sikokianin B and sikokianin C) were also obtained. Wikstroemia indica has been used to treat pneumonia, rheumatism, and bronchitis as folk medicine in China for long time. In 2012, the total synthesis of (+)-indicanone 454 was reported by Mukai and co-workers^286^ through the Rh(i)-catalyzed Pauson–Khand reaction of the allenyne derivative which was derived from (+)-limonene.

**Scheme 35:**
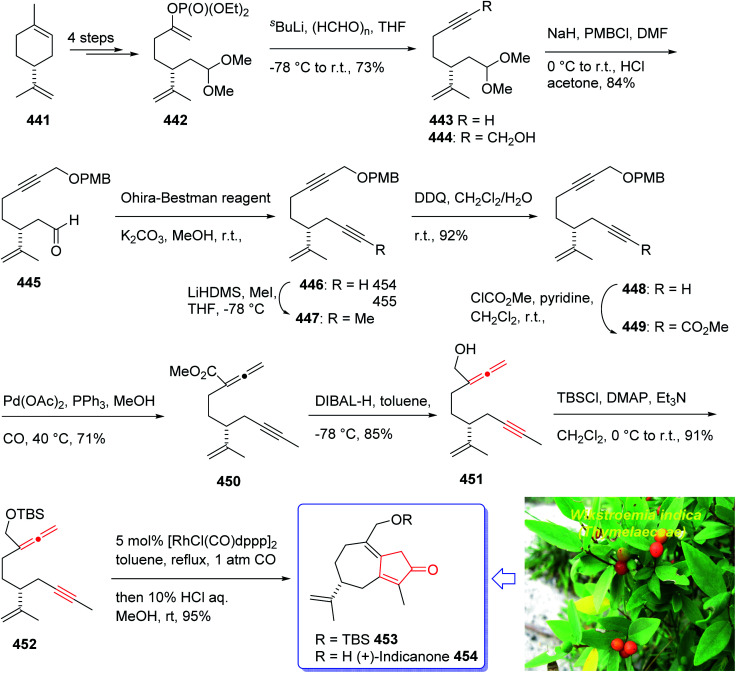
Total synthesis of (+)-indicanone 454.

In this strategy, the total synthesis of (+)-indicanone 441 commenced with vinyl phosphate 442,^287^ prepared from commercially available (+)-limonene after four steps. Compound 442 after several steps involving different common functional group transformations as well as protection–deprotection of some functional groups was converted to the allenyl alcohol of 451 which upon protection by a silyl group gave compound 452 as an appropriate precursor for PKR. The latter was subjected to PKR, upon treatment with 5 mol% [RhCl(CO)dppp]_2_ in toluene under reflux conditions and carbon monoxide (1 atm) supplied compound 453 in moderate yield. The latter was then desilylated using aqueous HCl gave the desired natural product (+)-indicanone 454. This strategy to the first total synthesis of (+)-indicanone 454 was completed in ten steps starting from easily accessible known phosphate 442 in 29% overall yield. In addition, this total synthesis confirmed the complete structure and absolute stereochemistry of (+)-indicanone 454, unambiguously (Scheme 35).^288^

In 2000, Fukuyama and his research group isolated merrilactone A 469, a complex cage-shaped pentacyclic sesquiterpene, from extract of *Illicium merrillianum*.^289^ Its structure was fully characterized showing it has an oxetane moiety, two γ-lactone functionalities, and a highly substituted cyclopentane ring at its core. In addition, merrilactone A 469 contains seven adjoining stereogenic centers, involving five quaternary ones. From the biological point of view, merrilactone A 469 is a nonpeptidal neurotrophic factor which stimulated neurite development in the values of fetal rat cortical neurons.^289^ Total synthesis of merrilactone A 469 has received much attention of organic synthetic chemists due to its exceptional and interesting structure as well as the reported activity against neurodegenerative diseases.^290^ Among them, Inoue and Hirama,^291–293^ Mehta,^294^ Frontier,^295^ Greaney^296^ and almost twenty years ago Danishefsky^290,297^ research groups have reported the total syntheses of merrilactone A 469. Other synthetic approaches have also been renowned for the total synthesis of related natural products to merrilactone A 469.^298–301^ Significantly, in 2012 Zhai and co-workers^302^ achieved and reported a new and proficient strategy to the total synthesis of (±)-469 involving the Pauson–Khand reaction (PKR)^58^ and hetero-Pauson–Khand reaction (h-PKR) in 2012.^303,304^ An effective total synthesis of (±)-merrilactone A was developed by recognition of an efficient installation of (+)-mintlactone through an intramolecular ynal h-PKR.^70^ It was commenced from the already reported alcohol 455,^305^ which upon treatment with triethyl orthopropionate and propionic acid provided the Johnson–Claisen rearrangement^306,307^ product 456 (dr = 3.8 : 1) that upon desilylation and lactonization produced compound 457 (dr = 2.9 : 1), 89% over two steps from 455 mediated by TsOH·H_2_O. Treatment of compound 457 with LDA, Ti(OiPr)_3_Cl, and then 3-trimethylsilylpropynal resulted in 1 : 1 mixture of inseparable aldols 458a and 458b in 81% combined yield alongside with two other inseparable isomers in 9% overall yield.^308^

**Scheme 36:**
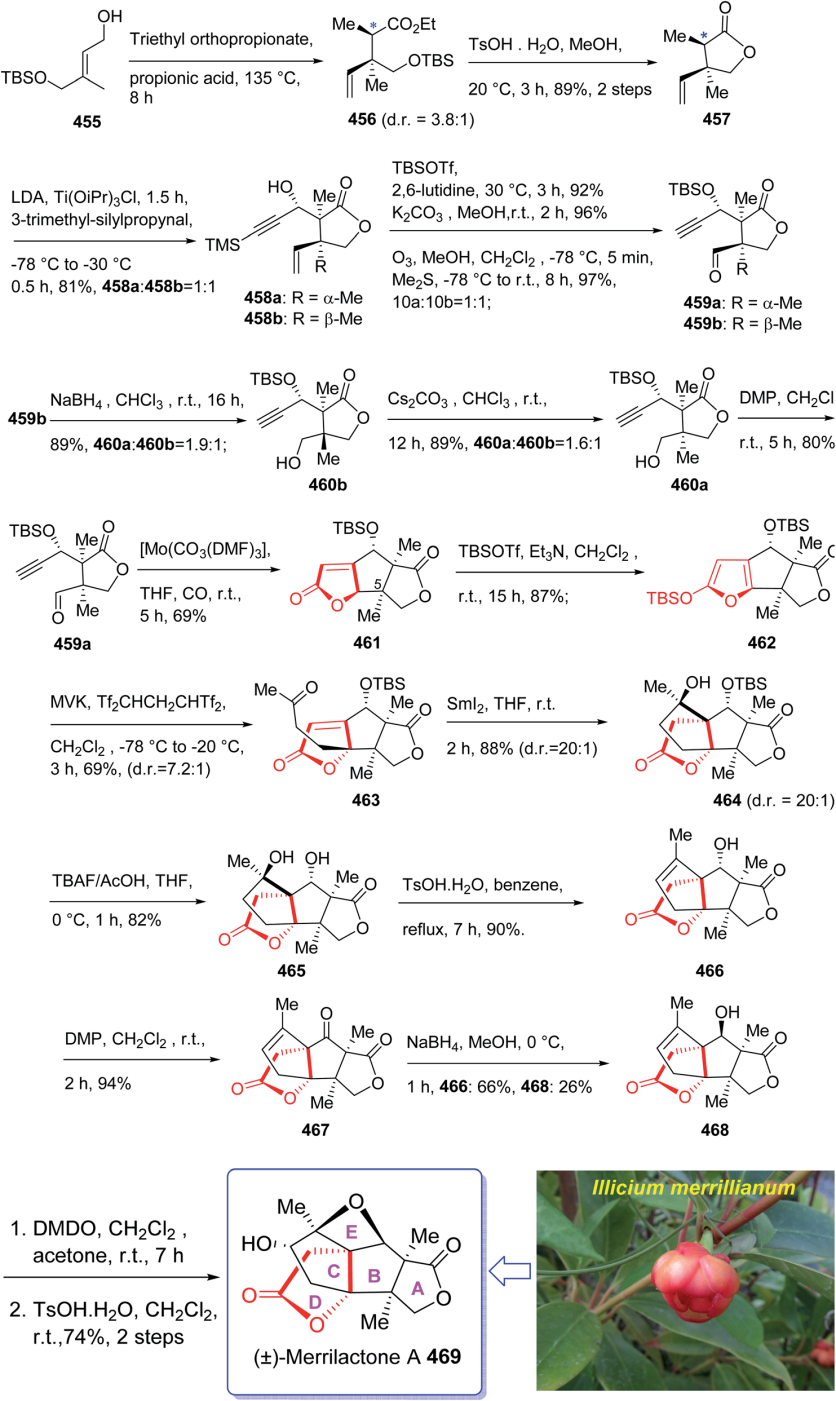
Total synthesis of merrilactone A 469.

Alcohols 458a and 458b were transformed smoothly into the two separable ynals 459a and 459b (1 : 1). Upon sequential hydroxyl protection, alkyne desilylation,^256^ and selective ozonolysis.^309^ Conversion of 459b into 459a was achieved *via* reduction of 459b using NaBH_4_ and intramolecular transesterification. Pleasantly, 460a was provided from 460b by initial transformation into a mixture of 460a and 460b (1.6 : 1) in the presence of Cs_2_CO_3_*via* intramolecular transesterification. Having ynal 461a available in hand, the vital h-PKR was performed to obtain the B and D rings of the target 468. The latter was treated with Mo(CO)_3_(DMF)_3_ (ref. 70 and 304) in THF at room temperature and under argon atmosphere provided tricycle 461 in 69% yield. Worthy to mention that 459a was not used up totally by replacing argon with carbon monoxide (CO). Pleasingly, when 459a was exposed to [Mo(CO)_3_(DMF)_3_] in THF at room temperature firstly under argon atmosphere for a while and then under CO atmosphere (balloon), compound 461 was obtained (69%). Then, α,β-unsaturated lactone 461 was transformed into silyloxyfuran 462,^310^ upon treatment with MVK mediated by Tf_2_CHCH_2_CHTf_2_ (ref. 311) under the Taguchi's strategy^311,312^ to give ketone 3 (61%) alongside with *epi*-3 (8%) *via* a vinylogous Mukaiyama/Michael addition reaction. Expectedly, the C ring could be constructed *via* a reductive carbonyl-alkene coupling reaction.^313,314^ Compound 463 was expectedly cyclized to furnish the desired tetracycle 464 (88%) as basically a simple diastereoisomer (dr = 20 : 1) upon treatment with SmI_2_ in THF. Reaction of the latter with TsOH·H_2_O in refluxing benzene with concurrent dehydration and desilylation resulted in the formation of the trisubstituted alkene 465 in 91% yield. On contrary, treatment of 464 with TBAF and AcOH instead of desilylation,^294^ gave diol 465 in 82% yield. Compound 465 upon dehydration in the presence of TsOH·H_2_O in refluxing benzene gave compound 466.^295^ Precisely, compound 466 by oxidation with DMP gave ketone 467 (94%) which after reduction with NaBH_4_ furnished an easily separable mixture of 468 (26%) and 466 (66%). This procedure was repeated many times to collect adequate quantities of alcohol 468. Ultimately, the latter was converted into the desired natural product, merrilactone, following a recognized procedure involving a stereoselective epoxidation and epoxide ring opening/oxetane generation (by homo-Payne rearrangement).^291,315^ The spectroscopic data of synthesized (±)-merrilactone A 469 was identical to those which already have reported for the natural product in the chemical literature.^289,290,294,295^ An efficient total synthesis of (±)-merrilactone A 469 has been reported in fifteen reaction steps for the shortest sequence from 7 which is a known compound (Scheme 36).^302,305^

**Scheme 37:**
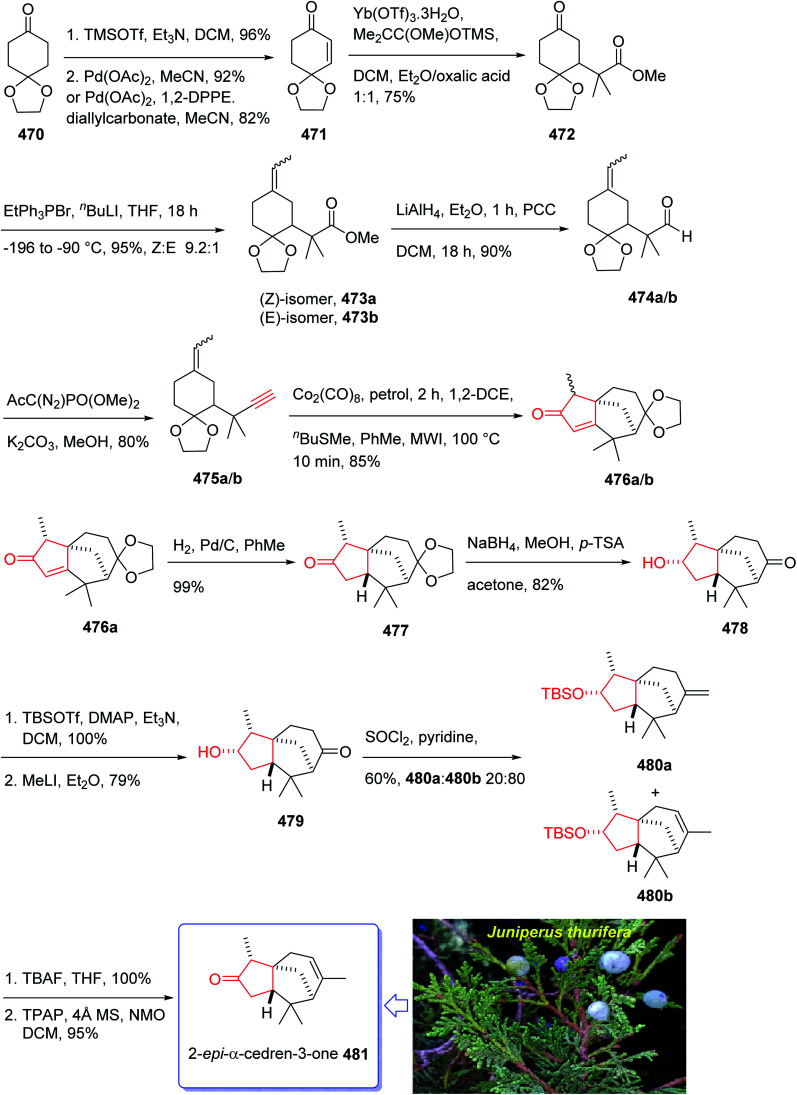
Total synthesis of 2-*epi*-α-cedren-3-one 481.

A sesquiterpene, 2-*epi*-α-cedren-3-one 481 is a natural compound, isolated from the essential oil of *Juniperus thurifera* in 2000 by Barrero and co-workers.^269^ Several efforts have been made to achieve its total synthesis due to its interesting molecular structure. A synthetic strategy was reported by Kerr and co-workers in 2018.^316^ involving a highly (*Z*)-selective Wittig olefination reaction. This reaction was performed at very low temperature which was essential to obtain of the same configuration within the desired natural product 481 and catalyzed intramolecular Pauson–Khand cyclisation reaction being conducted under MWI for the construction of the required tricyclic core of compound 481. This synthetic pathway started with market purchasable cyclohexanedione monoethylene acetal 470 which was converted to α,β-unsaturated ketone 471*via* Pd-catalyzed Saegusa oxidation reaction.^317^ The latter was then converted into ketoester 472*via* a conjugate addition of the silyl ketene acetal of methyl isobutyrate, mediated by ytterbium(iii) triflate trihydrate.^318^ Conversion of the latter to obtain the required alkene moiety is needed for (*Z*)-selective olefination reaction to attain the relative stereochemistry, more unambiguously being aligned with the methyl group *syn* to the methylene bridge in 2-*epi*-α-cedren-3-one, 481. Wittig olefination reaction was performed at ambient temperature to give olefins 473a and 473b in excellent yield. At this point, compounds 473a and 473b were found to be practically inseparable. Therefore, aldehydes 474a/b were provided *via* a two-step sequential reduction/oxidation. This mixture 474a/b was treated with the Ohira–Bestmann reagent^221,319^ (dimethyl acetyldiazomethylphosphonate) to obtain the alkyne 475a/b as suitable PKR precursors. Having enynes 475a/b available in hand, the effectiveness of the PKR, for the construction of tricyclic skeleton as core should have been optimized. To the purpose, addition of *n*-butyl methyl sulfide, as a promoter of PKR initially recognized by Sugihara and Yamaguchi,^320^ along with employing sub-stoichiometric quantities of Co_2_(CO)_8_ were successfully examined to obtain cyclopentenone 476a/b. It is worthwhile mentioning, as proved and reported earlier,^273^ the ratio of inseparable stereoisomers obtained *via* the Wittig olefination relates always directly to the ratio of cyclopentenone 476a/b provided in all PKRs reported previously. The mixture of 476a/b was converted into 480a and 480b after several steps. This mixture 480a/480b was separated and 480b was subjected to sequential deprotection and oxidation afforded the desired natural product 481 in 95% overall yield over two final steps. In conclusion, the total synthesis of 2-*epi*-α-cedren-3-one has been accomplished in seventeen steps using PKR as a vital step (Scheme 37).^316^

Pentalenolactone A 497 is a conspicuous member of naturally occurring antibiotics, generated by prokaryotic organisms.^321–324^ which was initially isolated and characterized as an acidic lipophilic antibiotic. The sesquiterpene antibiotic pentalenolactone 497, isolated from a variety of *Streptomyces species*, is a rare example of a cyclic terpenoid produced by a prokaryotic organism. Following the original isolation in 1957 by Koe and co-workers, McBride and co-workers,^324,325^ the structure and absolute configuration were eventually assigned in 1970 by combination of spectroscopic and X-ray crystallographic methods.^326,327^ The structure of pentalenolactone A 497 was determined by NMR analysis of its methyl ester derivative. The analysis showed that the structure consists of a highly compact carbon framework with a rare, angularly fused tricyclic pentanoid lactone having various oxidation states in each of the rings. It has a tricyclic core showing cytotoxic activity.^328,329^ Such compounds also show a broad spectrum of potencies towards bacteria, fungi, viruses, and tumors. Its stimulating properties and substantial chemical activity prompted Danishefsky *et al.* to develop a fascinating total synthesis of 497 in 1978.^330–332^

**Scheme 38:**
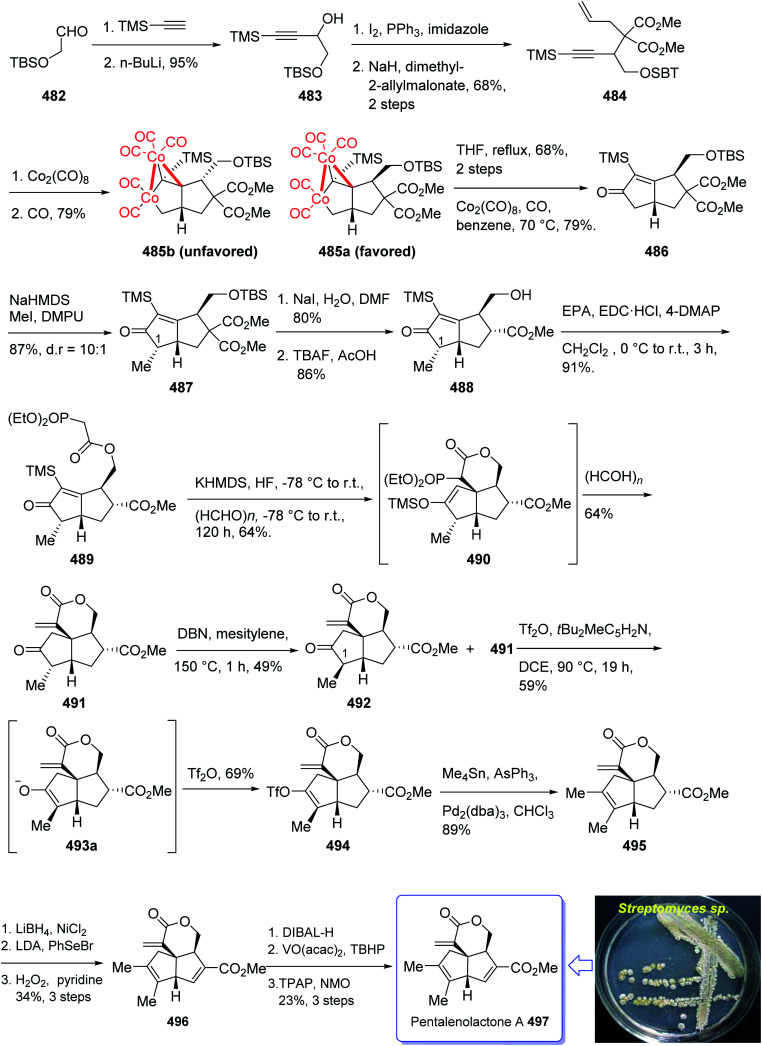
Total synthesis of pentalenolactone A 497.

In 2012, Li and co-workers^332^ reported a protocol^333,334^ for the asymmetric total synthesis of methyl ester of pentalenolactone A 497 based on an intramolecular Pauson–Khand reaction. It began with aldehyde 493 which upon treatment with lithium ethynylate at −78 °C afforded propargyl alcohol 483 in 95% yield. The newly formed hydroxyl group in 483 was transformed into the respective iodo compound upon treatment with I_2_/imidazole mediated by Ph_3_P and the resulting iodo species was next reacted with sodium dimethyl 2-allylmalonate to furnish the enyne 484 in 68% yield in two steps as an ideal precursor for the PKR. The latter was then converted to cyclopetenone 486*via* PKR. The exclusive construction of 486 was most probably to minimize the steric interaction between the TMS and CH_2_OTBS (TBS = *tert*-butyldimethylsilyl) moieties in complex 485a^53,247^ relative to complex 485b, which consequently resulted in the formation of the desired annulated product 486 as the sole product. Having the latter available in hand, product 496 in an overall yield of 34% was converted to the desired natural product methyl ester of pentalenolactone A 497 after several steps following the already reported procedure by Danishefsky and co-workers in 1978 (Scheme 38).^332,335^

**Scheme 39:**
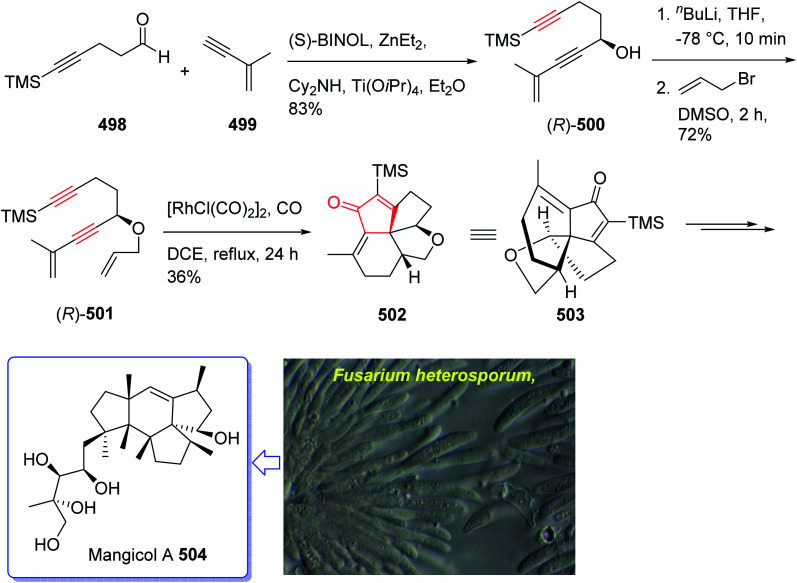
Total synthesis of mangicol A 504.

In 2000, Fenical research group isolated Mangicol A 504 (ref. 336) from a marine fungal *Fusarium heterosporum*. They reported it as a novel type of sesterterpene polyols with an exceptional spirotricyclic scaffold bearing a quaternary chiral carbon core.^336^ Mangicol A 504, among the other members of this class of naturally occurring compounds, has been found to be active anti-inflammatory agents, thus, has important therapeutic effect. Many unfinished efforts have been made for the total synthesis of this kind of compounds.^337–340^ For instance, Uemura and co-workers in 2004 reported the total synthesis of core structure of mangicols A 504 in twenty-nine steps employing a Diels–Alder reaction of a macrocycle.^337^ In 2012, Yu and co-workers^14,15,56,58,341^ designed and effective pathway to obtain the spirotricyclic core analogue of mangicol A. This route involved asymmetric Pauson–Khand reaction and intramolecular Diels–Alder reaction as key steps. The total synthesis began from the BINOL-based catalytic asymmetric addition of enynal 498 to enyne 499 leading to formation of the optically active propargylic alcohol (*R*)-500. Treatment of the latter with *n*-BuLi followed by reaction with allyl bromide in DMSO afforded the corresponding diene–diyne substrate (*R*)-501 as an appropriate precursor for PKR. Thus, the latter was subjected to the rhodium(i)-based catalyst and then to the PKR conditions ([RhCl(CO)_2_]_2_, CO, reflux, 24 h). The two cycloadditions of alkyne units took place to furnish compound 502. The latter was converted after several steps to the desired natural product, the mangicol A 504 (Scheme 39).^342^

### 2.5. Sesquiterpenoids

**Scheme 40:**
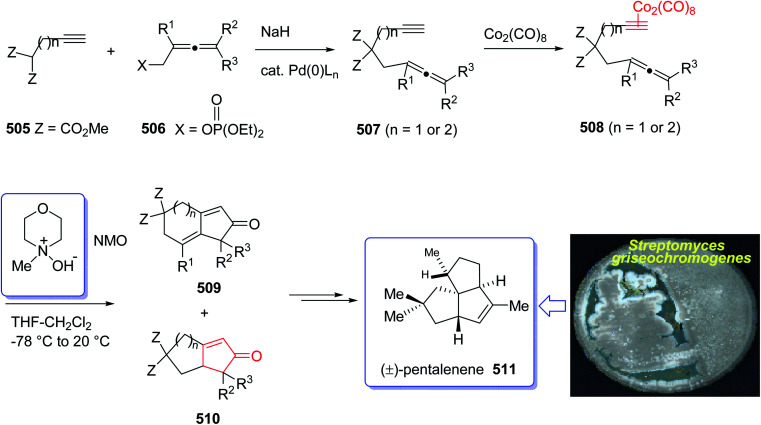
Total synthesis of (±)-pentalenene 511.

Piers *et al.*^343^ in 1984 reported total synthesis of (±)-pentalenene 511 using Pauson–Khand reaction. The natural product (±)-pentalenene 511, belongs to the pentalenolactone family of sesquiterpenoid antibiotics. It was initially isolated in 1980 by Seto and Yonehara from *Streptomyces griseochromogenes*.^244^ It has an interesting structure containing the parent hydrocarbon. In fact, (±)-pentalenene 511 has been found^344^ to be a biosynthetic precursor of pentalenolactone.^327,345,346^ Initially, several α,ω-allenynes 507 [1,2-dien-7-ynes 507a–d (*n* = 1) and 1,2-dien-8-ynes 507e,f (*n* = 2)] which were requisite for this total synthesis were provided *via* the Pd-catalyzed reaction of enyne 505 with the phosphates 506 in the presence of NaH in which the phosphate moiety was substituted. Unambiguously, cyclization of the bicyclic enone *via* the tertiary amine oxide-promoted PKR of enyne 507 was anticipated to proceed in the expected desired stereochemical sense. Consequently, intramolecular cobalt-catalyzed PKR of α,ω-allenynes 507 as an appropriate PKR precursor resulted in the formation of bicyclic dienones 509 and α-alkylidenecyclopentenones 511, regioselectively, *via* formation and conversion of intermediate hexacarbonyldicobalt complex 508a depending on the substitution pattern of the allenic moiety. It seemed likely that transformation of α-alkylidenecyclopentenones 509 into (±)-511 after several steps is straightforward leading to the formation of the desired natural product (±)-pentalenene 511 (Scheme 40).^343,347^

Paecilomycine A 523, is a tricothecane derived naturally occurring sesquiterpenoid which was initially isolated in 2004 by Oshima and co-workers, among other *Paecilomyces tenuipes* and terpenoids from extract of cultured fruiting bodies of *Paecilomyces tenuipes* (*Isaria japonica*).^126,348^ Paecilomycine A 523 showed an increased and inspiring neurite outgrowth in PC-12 cells and impressive neurotrophic activity.^126^ The total synthesis of paecilomycine A 523 was reported by Mehta *et al.* in 2012 using intramolecular Pauson–Khand reaction.^349^ This strategy commenced with ethyl 4-methyl-2-oxocyclohex-3-enecarboxylate 512 which was submitted to propargyloxy-methylation using (propargyloxy) methyl chloride^350^ to give 513. The latter was subjected to stereoselective Luche reduction^351^ in compound 513 to afford α-hydroxy compound 514. The latter then after several steps including protection–deprotection and various functional group transformations such as Wittig olefination, was converted to 518 as an appropriate PKR precursor. In a key step, the latter underwent stereoselective intramolecular PKR in the presence of Co_2_(CO)_8_ and NMO in CH_2_Cl_2_ to provide the spiro-fused tricyclic hydroxy-enone 519.

**Scheme 41:**
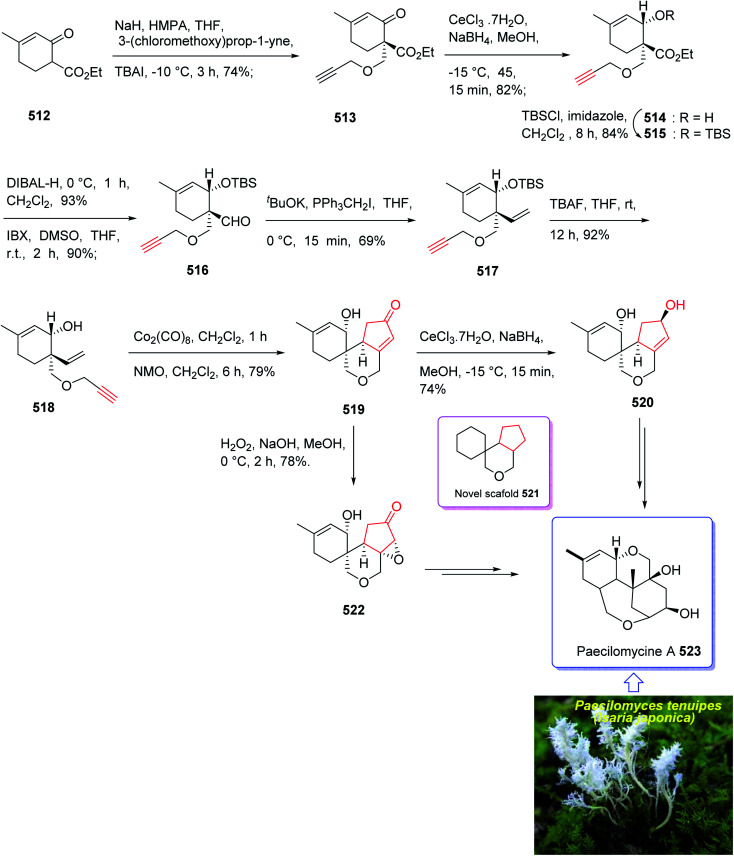
Total synthesis of paecilomycine A 523.

As a matter of fact, two derivatives were synthesized from 519: (a) a tricyclic diol 520 obtained from stereoselective Luche reduction and (b) the respective epoxide 521 by nucleophilic epoxidation which was used for biological screening. Remarkably, all of the novel synthesized compounds 519, 520, 522 symbolizing the 2-oxa-spiro[5.5]undecane segment, which were already recognized to be neuroprotective in standard MTT and trypan blue for cell viability screening.^352–354^ Interestingly, both compounds, 520 and 522 were converted to the desired natural product paecilomycine A 523. The expedition for the synthesis of novel structure exhibiting neurotrophic activity, encouraged by paecilomycine A 523, has resulted in the design and synthesis of a novel framework containing 2-oxa-spiro [5.5] undecane core (Scheme 41).^349^

Among terpenes as a wide-spread family of naturally occurring small compounds, sesterterpenoids probably are the most enthralling molecules with complicated architectures showing various biological potencies. Several members showed anticarcinogenic, antimicrobial, anti-inflammatory, and cytotoxic potencies.^355,356^ One of these sesterterpenoids found to have a compound so-called astellatol 544.^357,358^ Initially, in 1989 Sadler and co-workers isolated astellatol 544 from extract of *Aspergillus stellatus* and elucidated its structure.^357^ According to their structural determination, the left part of astellatol 544 structure possesses a crowded ring system with several stereogenic centers involving quaternary centers which makes its synthesis difficult. In addition, another major synthetic problem is the presence of highly substituted, notorious *trans*-hydrindane moiety on the right side.^359,360^ Astellatol 544 possesses an executional bicycle [4.1.1] octane moiety, ten stereogenic centers, a cyclobutane involving two quaternary centers, an *exo*-methylene group, and a sterically hindered isopropyl *trans*-hydrindane moiety. Due to the structural complexity of astellatol 544, its total synthesis has been very challenging and stimulating which took about 30 years to be successfully achieved.

**Scheme 42:**
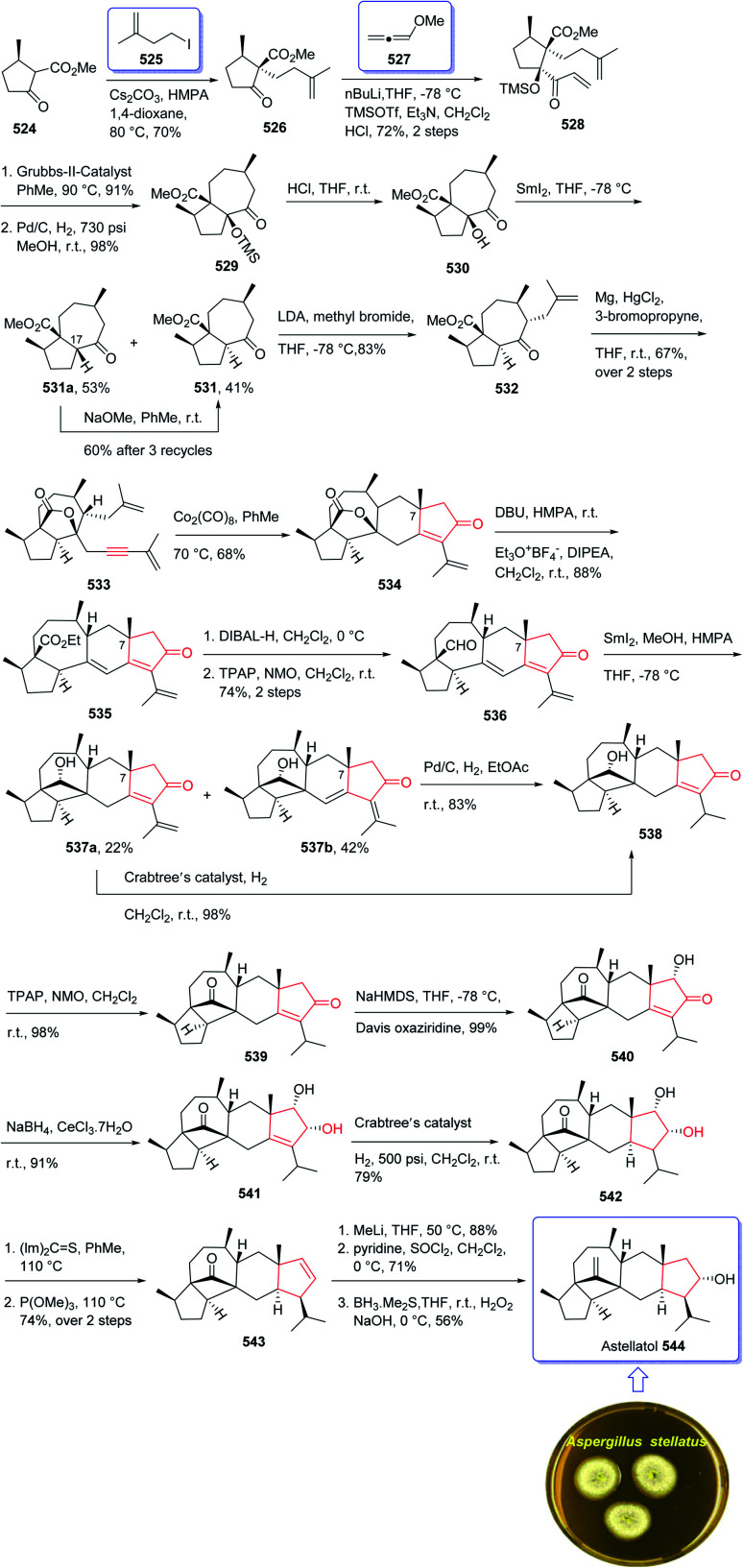
Total synthesis of astellatol 544.

The total synthesis of astellatol 544 was successfully attempted by E. J. Corey and co-workers in 1985,^360^ and later by Paquette,^361,362^ Hudlicky,^363,364^ and Wender.^365^ In 2018, Xu *et al.* also accomplished 536 (ref. 366) and reported a brief and asymmetric synthesis of astellatol 544. That was including a SmI_2_-catalyzed reductive radical 1,4-addition.^367^ This synthetic pathway started from the alkylation of the already reported chiral synthon 524 (ref. 368) with the homoallylic iodide 525 in the presence of Cs_2_CO_3_ and HPMA in dioxane under reflux to provide ketone 526. The latter was attacked by the lithiated derivative of methoxypropadiene 527,^369^ which upon protection and hydrolysis gave the enone 528. The latter after several steps was transformed to multigram quantities of 533, as an appropriate precursor for PKR. Pleasantly, the substrate 533 underwent PKR using Co_2_(CO)_8_, in 2015 by Yang *et al.*^370^ to afford the expected cyclized product 534. After several steps, compound 534 was converted to alkene 543. Then, the latter was converted to the desired natural product astellatol 544 in two steps. In the first step, alkene 543 was reacted with MeLi at 50 °C to give the tertiary alcohol, which successfully subjected to elimination in the presence of SOCl_2_/pyridine conditions which leading to the *exo*-methylene functionality on the cyclobutane framework. In the second step, a hydroboration-oxidation occurred to give astellatol 544 in 56% yield. As a result, the total synthesis of the rare sesterterpenoid, astellatol 544, was achieved in twenty-five steps and 0.63% overall yield starting from chiral synthon 524 (Scheme 42).^366,371^

**Scheme 43:**
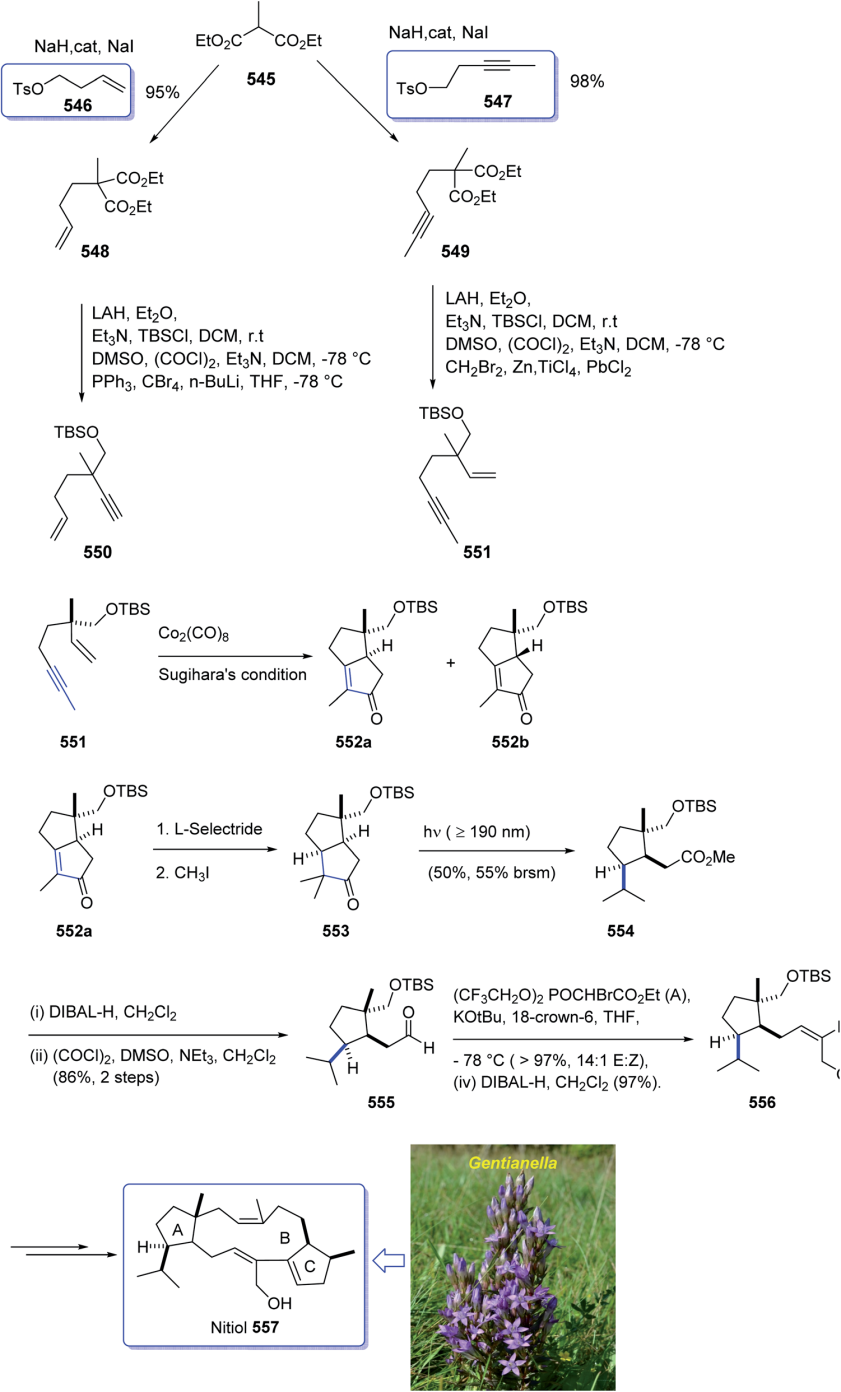
Total synthesis of A-ring component of nitiol 557.

A concise and asymmetric total synthesis of A-ring component of nitiol 559 including diastereoselective Pauson–Khand cycloaddition was reported by Dake in 2001.^372^ It was actually an attempt towards the total synthesis of the natural product, nitiol 558.^105,360,373^ In 1995, Cordell and co-workers found out that the plant so-called *Gentianella* (G.) is a regular remedial plant that cultivates in the Andes area. Its extract contains several compounds with diverse structures showing various biological activities, extensively employed as folk medicine for the treatment of hepatitis, and obesity.^374,375^ Nitiol 558 was initially extracted from this whole plant and was separated several times and purified *via* different techniques by Kawahara research group in 1999 resulting in the isolation of, novel sesterterpenoid.^376^ Interestingly, its structural elucidation was made by the same group in the same year.^376^ Primary biological screening showed that compound 558 behaves as an active enhancer of interleukin-2 in human T cell lines.^376^ The total synthesis of nitiol 558 was accomplished and reported by Dake and co-workers in 2001.^372^ The important steps in this strategy are an asymmetric Pauson–Khand cycloaddition and a norrish type 1 fragmentation reaction.^105,360,373^ The total synthesis started from diethyl methylmalonate 545 which upon alkylation with the appropriate tosylate in the presence of sodium hydride and catalytic amount of sodium iodide in DMF gave 2,2-disubstituted malonates 548 and 549.^377,378^ The required enyne 551 and 550 were prepared *via* standard procedures involving, LAH reduction, TBSCl protection, oxidation, Corey–Fuchs or Nozaki reactions. Compound 551 was then subjected to Sugihara's conditions^15,379,380^ to provide desired bicyclooctenes 552a and 552b in satisfactory yields (57–74%). The prepared cyclopentenone 552a then underwent conjugate reduction by utilization of lithium tri(*sec*-butyl) borohydride followed by quenching with methyl iodide to afford 553 in high isolated yield in pure form. Delightfully, this process afforded trisubstituted cyclopentane 554 in 50% yield. In this step, the ester functional group of 554 was transformed into an aldehyde 555 under typical conditions, in two steps including reduction using diisobutylaluminum hydride and Moffatt–Swern oxidation in 86% overall yield. The latter upon treatment with Kogen's Horner-Wadsworth-Emmons-type reagent A^381^ gave the tri-substituted vinyl bromide 556 in excellent yield (>97%) and high stereoselectivity (*E* : *Z*, 14 : 1). Finally, the latter after several steps was converted to the desired natural products 557 in 40% overall yield (Scheme 43).^372^

## 3. Conclusion

In conclusion, in this review, we tried to draw the attention of readers, especially synthetic organic chemists to one of the most useful name reactions in organic chemistry so-called Pauson–Khand reaction (PKR). We presented a brief summary of the catalytic PKR. As we mentioned there are just a few organic transformations that complement so much molecular complexity in one step as the PKR. The products of PKRs are cyclopentenones, which can be readily converted into various functionalized cyclopentanes, which exist as structural elements in the scaffolds of several natural products. Then, we discussed the merits and drawbacks, observed for PKR, during the years. As mentioned, the intramolecular PKR (IPKR) was developed. In addition, various transition metal catalysts were introduced. We showed that by using modified (chiral) catalysts and reaction conditions, the products of PKR can be synthesized, asymmetrically. Thus the asymmetric variant of this important name reaction, nowadays is quite possible, leading to optically active products. Asymmetric PKRs can be achieved by using different approaches, including use of chiral ligands and chiral metal complexes. Comprehension of the reaction mechanism will allow accurate prediction of the stereochemical outcome of the reaction. Thus, we focused on the applications of PKR in the total synthesis of natural products trying to encourage synthetic chemists to rely on this reaction when designing their synthetic pathways leading to total synthesis of an appropriate natural products. In addition, the content of this review has been arranged based on the family and types of plants, which the certain natural product has been isolated from and their biological activities were also mentioned. That makes this review in addition to organic synthetic chemists, useful and readable to natural products chemists, pharmacists and botanists.

## Conflicts of interest

There are no conflicts to declare.

## Supplementary Material
